# Chromium Flavonoid Complexation in an Antioxidant Capacity Role

**DOI:** 10.3390/ijms23137171

**Published:** 2022-06-28

**Authors:** Sevasti Matsia, Olga Tsave, Antonios Hatzidimitriou, Athanasios Salifoglou

**Affiliations:** 1 Laboratory of Inorganic Chemistry and Advanced Materials, School of Chemical Engineering, Aristotle University of Thessaloniki, 54124 Thessaloniki, Greece; srmatsia@cheng.auth.gr (S.M.); tsaveolga@auth.gr (O.T.); 2 Laboratory of Inorganic Chemistry, School of Chemistry, Aristotle University of Thessaloniki, 54124 Thessaloniki, Greece; hatzidim@chem.auth.gr

**Keywords:** chromium complexation, flavonoid chrysin, oxidative stress, antioxidant capacity, myoblasts, structure-function correlation, antiradical activity

## Abstract

The plethora of flavonoid antioxidants in plant organisms, widespread in nature, and the appropriate metal ions known for their influence on biological processes constitute the crux of investigations toward the development of preventive metallodrugs and therapeutics in several human pathophysiologies. To that end, driven by the need to enhance the structural and (bio)chemical attributes of the flavonoid chrysin, as a metal ion complexation agent, thereby rendering it bioavailable toward oxidative stress, synthetic efforts in our lab targeted ternary Cr(III)-chrysin species in the presence of auxiliary aromatic N,N′-chelators. The crystalline metal-organic Cr(III)-chrysin-L (L = bipyridine (**1**) and phenanthroline (**2**)) compounds that arose were physicochemically characterized by elemental analysis, FT-IR, UV-Visible, ESI-MS, luminescence, and X-ray crystallography. The properties of these compounds in a solid state and in solution formulate a well-defined profile for the two species, thereby justifying their further use in biological experiments, intimately related to cellular processes on oxidative stress. Experiments in C2C12 myoblasts at the cellular level (a) focus on the antioxidant capacity of the Cr(III)-complexed flavonoids, emphasizing their distinct antiradical activity under oxidative stress conditions, and (b) exemplify the importance of structural speciation in Cr(III)-flavonoid interactions, thereby formulating correlations with the antioxidant activity of a bioavailable flavonoid toward cellular pathophysiologies, collectively supporting flavonoid introduction in new metallo-therapeutics.

## 1. Introduction

Flavonoids, as large naturally occurring organic molecules, belong to ubiquitously encountered families of natural products, with important roles in plant biochemistry and physiology, higher animals, and humans [1]. The multitude of environmental pressures exerted on an equally diverse and plethoric plant kingdom signifies the tremendous number of flavonoids arisen through the ages to protect plants in their microcosmic environment and provide interjecting mechanistic pathways with molecular agents contributing to normal physiology and survival [2]. They possess a wide variety of biological activities, including antithrombotic, antibacterial, antiproliferative, antifungal, antiviral, anti-inflammatory, antioxidant, and antidiabetic potential [3,4,5]. They also act as enzyme inhibitors and are involved in photosensitization and energy transfer [6,7]. Certain flavonoids also function as plant growth hormones and growth regulators. They are involved in the control of respiration, photosynthesis, morphogenesis, and sex determination, as well as defense against infections [8,9,10]. Despite the fact that they are found in the plant kingdom and are isolated through extraction techniques, there has been much research work done to increase their production using bacterial species, i.e., *Streptomyces albus*, targeting apigenin, kaempferol, luteolin, eriodictyol, and quercetin [11,12,13,14].

Given the hitherto known biological activity of flavonoids as phytochemicals in plant physiology, their introduction in treatments of pathological conditions in humans has attracted keen interest over time. Such efforts have progressively followed the path of medical therapeutics from traditional folk medicine (of thousands of years of history) to contemporary pharmaceuticals, thereby involving such compounds as molecular precursor species to therapeutics against metabolic diseases. In such a view, focus on specific flavonoids and their role in metabolism-related diseases in humans appears to provide a significant impetus toward the synthesis of new drugs, including metallodrugs. To that end, the established role of flavonoids in biomimesis (for instance, insulin-like effects) stands as the foreground for launching synthetic efforts toward new enhanced drugs with specificity to the target for which they are intended [15,16,17,18].

**Scheme 1:**
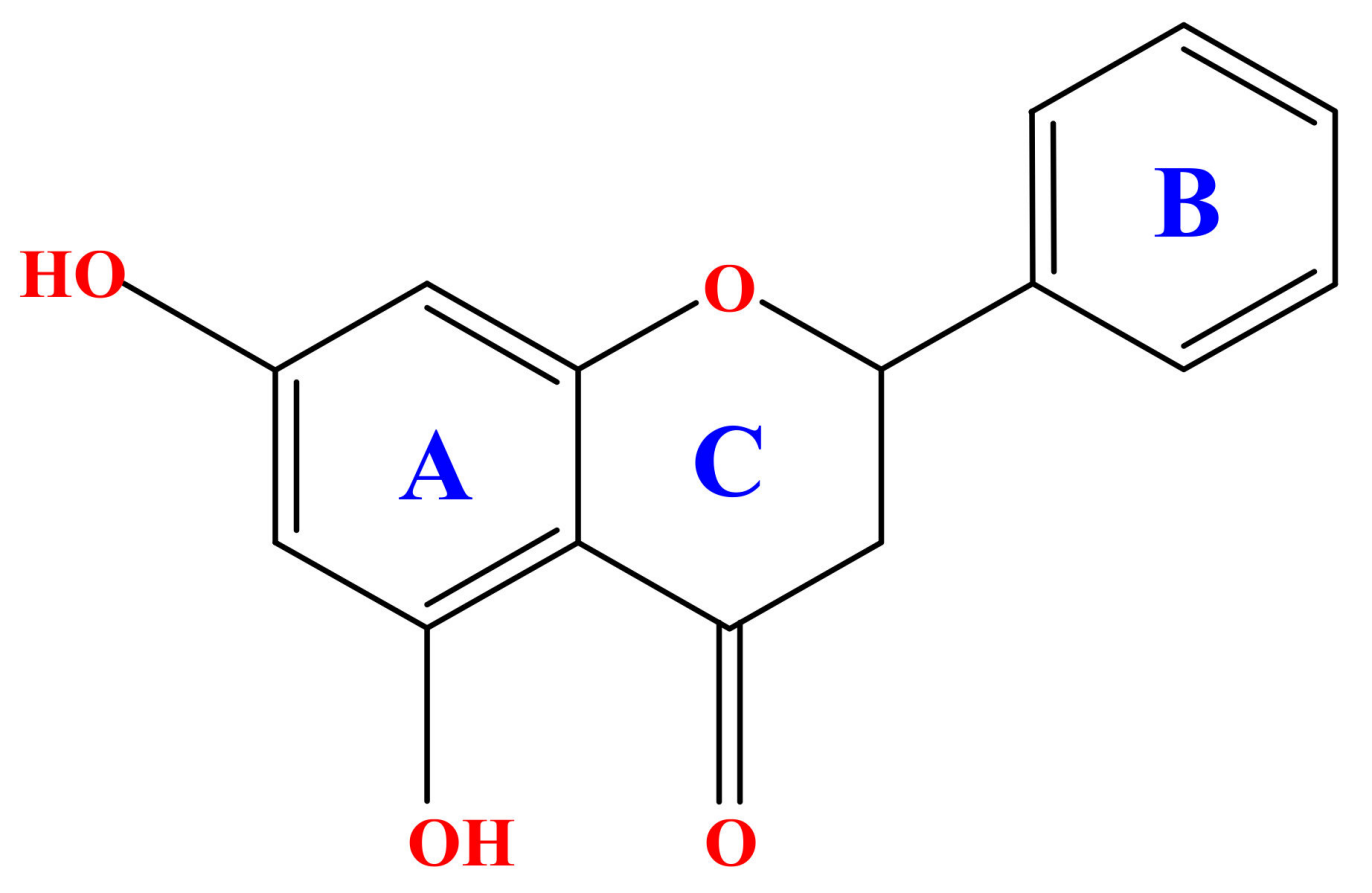
Core structure of a flavonoid exemplified into the molecular structure of chrysin.

One such naturally encountered flavonoid is chrysin (Scheme 1), known for its multiple beneficial actions. Chrysin has been shown to activate antioxidant enzymes and regulate glutathione levels. Moreover, chrysin can restore mitochondrial dysfunction in oxidative stress-related pathological conditions (e.g., diabetes mellitus, Alzheimer’s disease, and myopathies) [19], alleviating secondary complications including neuropathy, retinopathy, and cardiomyopathy, among others [20,21]. Significant problems, however, arise with the effectiveness of its biological activity owing to the limited solubility and bioavailability, thereby necessitating the adoption of new approaches enhancing its access to molecular targets and efficacy as an antioxidant potency carrier. One such approach resorts to metal ion complexation as a means of improving the (bio)chemical attributes of chrysin (and other flavonoids) in a biological environment.

Metal ions have since long been used in the treatment of human diseases, with a diverse spectrum of compounds involving both metal ions and appropriately configured (in)organic ligand-substrates. To that end, unique combinations of both inorganic and organic nature into a more potent form of a drug strive to deliver a therapeutic load to a tissue target with varying and broad specificity. Metallodrugs have merit at both the diagnostic and therapeutic levels, and they constitute a chemical and biological challenge in that they aspire to alternative, improved, and potentially more effective agents in contemporary clinical disease administration. Among the metal ions involved in such drugs, selected forms of Pt, Au, Bi, Cu, Zn, and Cr have over the years been formulated and are presently employed as metallodrugs. As a representative metal ion present in metallodrugs, Cr(III) has been one of the conjectured species, with binary compounds thereof being available as medicaments, contributing to the improvement of symptomatologies in diabetes mellitus [22,23,24].

Promoting the emergence of new alternative metallodrugs, naturally occurring organic metabolites, such as flavonoids, known for their antioxidant potency and properties, stand as competent precursors of metal-organic compounds of therapeutic potential. Being aware of the importance of the chemistry of flavonoids in nature and thus their potential chemistry with metabolically relevant metal ions, such as Cr(III), in generating new metal-organic compounds, efforts were launched in our lab to investigate synthetically ternary systems of that trivalent metal ion with flavonoid chrysin and N,N′-organic aromatic chelators, such as 2,2′-bipyridine (bipy) and 1,10-phenanthroline (phen). Such well-defined ternary complex Cr(III)-flavonoid species were synthesized and characterized crystallographically for the first time, justifying further use in biological investigations. The ensuing investigation of the biological activity of the arising hybrid Cr(III)-flavonoid compounds comes from the fact that flavonoids themselves exhibit firmly antioxidant activity properties, effectively counteracting any ROS-emerging action in sensitive tissues in humans. Consequently, flavonoid incorporation into the coordination sphere of metal ions appears to formulate attractive new drug forms or systems, which may not only enhance metal-linked drug activity but also maintain ROS homeostasis over time in response to the amount of ROS present or rising into a cellular system. To that end, in the present study, the in vitro model of C2C12 myoblasts was employed to further investigate the antioxidant capacity of the newly synthesized complexes [14]. The collective chemical and in vitro biological investigation efforts aim at highlighting the contribution of well-defined metalloforms of natural flavonoids (structural speciation) (a) toward the enhancement of their bioavailability through metal ion complexation, and (b) to the therapeutic arsenal in the pathology of oxidative stress, thereby meriting the development of metal-flavonoid drugs in human health and disease.

## 2. Results

### 2.1. Syntheses

Both ternary Cr(III)-chr-bipy (**1**) and Cr(III)-chr-phen compounds (**2**) were synthesized under aerobic conditions through simple reactivity approaches, involving the appropriate reagents of interest, followed by the vapor diffusion of appropriate precipitating solvents for their isolation in crystalline form.

**Scheme 2:**
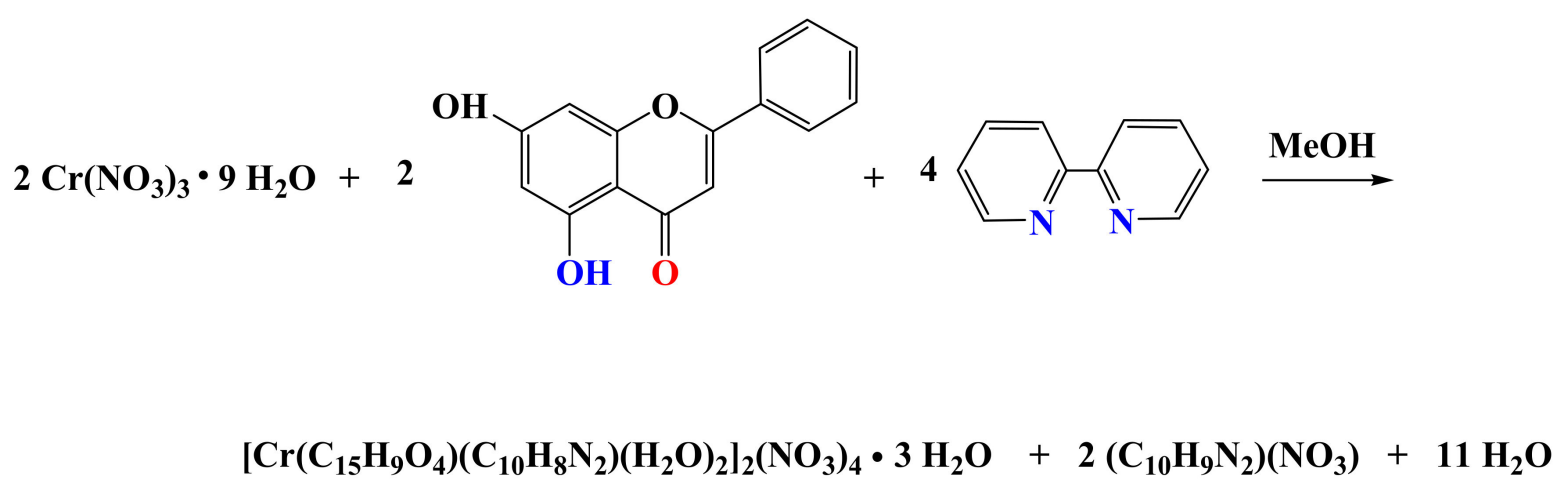
Stoichiometric reaction leading to the synthesis of **1**.

**Scheme 3:**
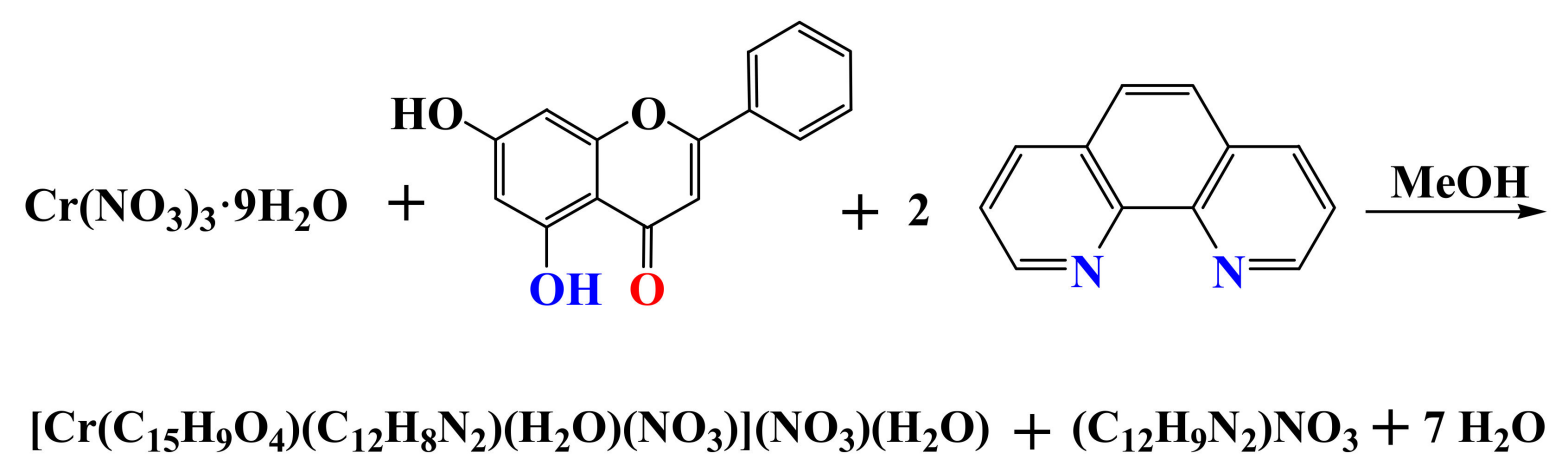
Stoichiometric reaction leading to the synthesis of **2**.

Specifically, compound **1** was expediently synthesized through a simple reaction in a procedure developed in our lab. Specifically, the reaction of simple reagents Cr(NO_3_)_3_ 9 H_2_O, chr, and bipy in methanol led to the isolation of a single crystalline material at the bottom of the vial. Compound **1** was identified through (a) elemental analysis, (b) the FT-IR spectrum, and (c) the X-ray crystal structure determination of one of the isolated single crystals of the metal-organic material. The overall stoichiometric reaction leading to **1** is shown below (Scheme 2):

Compound **2** was synthesized through the reaction of Cr(NO_3_)_3_·9H_2_O, chr, and phen in methanol. The isolated crystalline material, collected after vapor diffusion, was identified through (a) elemental analysis, (b) the FT-IR spectrum, and (c) the X-ray crystal structure determination. The stoichiometric reaction leading to **2** is shown below (Scheme 3):

The materials obtained through the specific approach were stable at room temperature in the open air. Freshly prepared compounds **1** and **2** were soluble in water (at low mM concentrations), methanol, and DMSO, thus allowing further analysis through UV-Visible, ESI-MS, and photoluminescence.

### 2.2. Description of X-ray Crystallographic Structures

The X-ray crystal structure determination of the new materials revealed that both compounds consist of discrete anionic and cationic moieties. Selected bond lengths and angles of the complexes are presented in Table 1.

**Figure 1:**
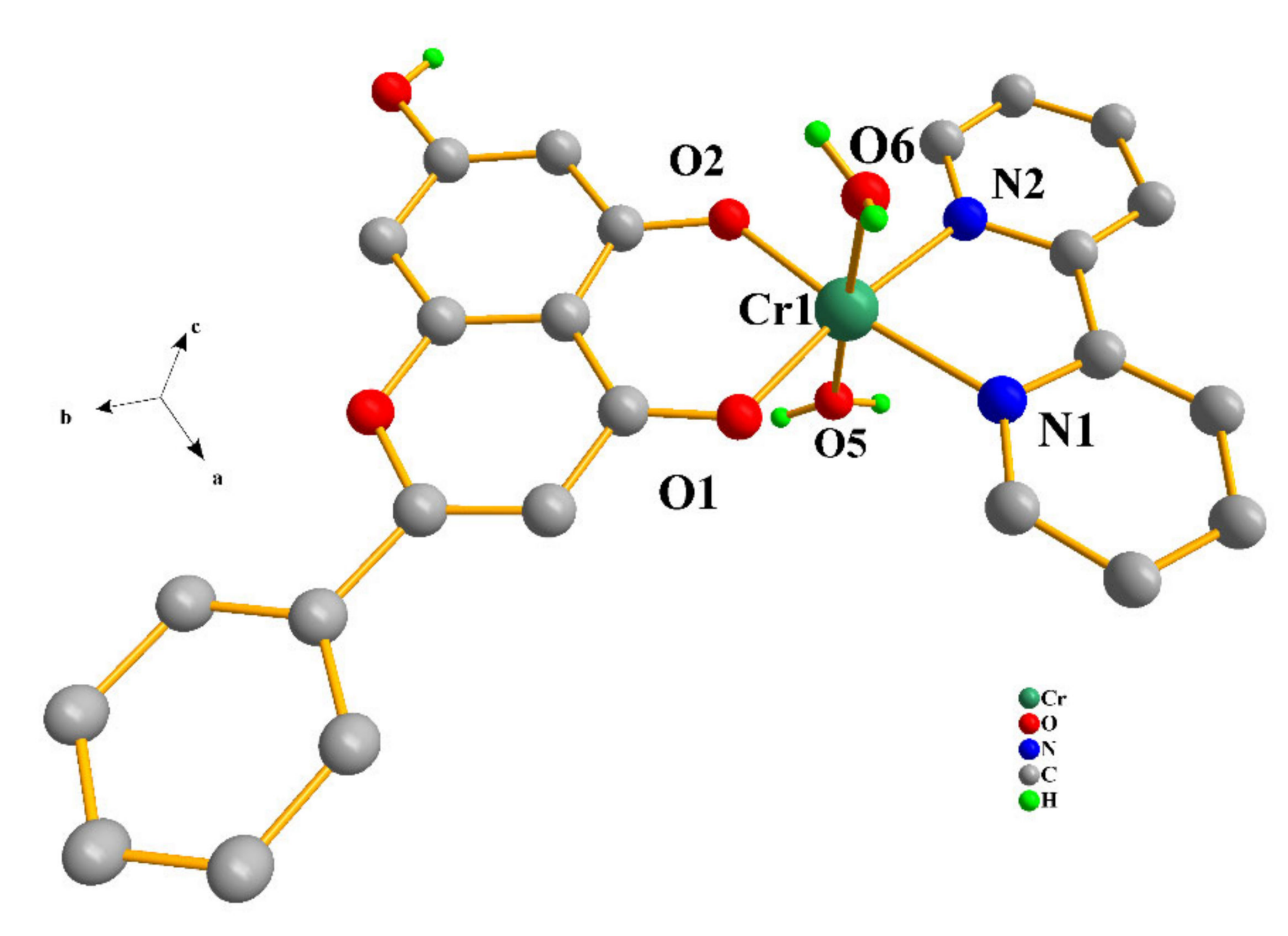
Diamond diagram of the cationic assembly [Cr(C_15_H_9_O_4_)(C_10_H_8_N_2_)(H_2_O)_2_]^2+^ in compound **1**. Hydrogen atoms connected to sp^2^ carbons are omitted.

Compound **1** crystallizes in the triclinic space group *P*ī, with two complex formula assemblies in the unit cell. The cell consists of two dicationic complex assemblies [Cr(C_15_H_9_O_4_)(C_10_H_8_N_2_)(H_2_O)_2_]^2+^, four nitrate counter ions, and three lattice water molecules (Figure 1). The metal ionic assembly involves a central Cr(III) metal ion surrounded by one chrysin and one bipyridine chelator. The chrysin ligand binds the central metal ion through the keto moiety of the C ring and the abutting phenolato moiety of the A ring. Deprotonation of the chrysin ligand at the A ring phenolic terminal and binding of the metal ion through the phenolato moiety generate a six-membered metallacyclic ring, thus contributing to the stability of the entire assembly. In an analogous fashion, the bipyridine chelator binds the metal center through the sp^2^ nitrogen anchors, further stabilizing the metal assembly through a second five-membered metallacyclic ring. Two water molecules also bind the metal ion at diametrically opposed positions. Therefore, the overall coordination geometry around Cr(III) is six-coordinate distorted octahedral. In the so-formed octahedron, the two oxygen atoms of chrysin and the two nitrogen atoms of the bipyridine ligand occupy the equatorial plane, whereas the oxygen atoms from the aqua ligands occupy the axial positions.

**Figure 2:**
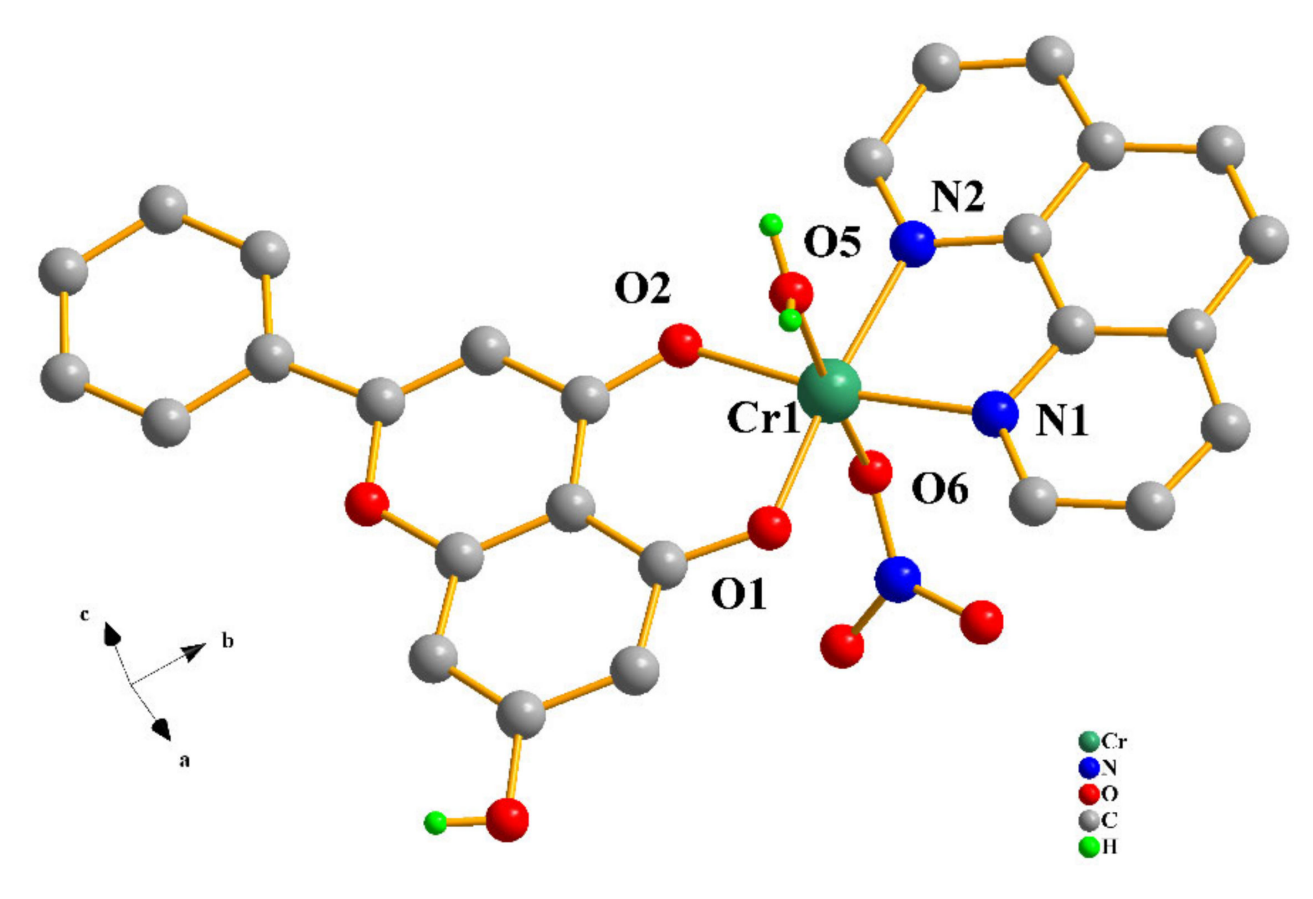
Diamond diagram of the cationic assembly [Cr(C_15_H_9_O_4_)(C_12_H_8_N_2_)(H_2_O)(NO_3_)]^+^ in compound **2**. Hydrogen atoms connected to sp^2^ carbons are omitted.

Compound **2** crystallizes in the monoclinic space group *P*2_1_/*n*, with four formula moieties in the unit cell. The asymmetric unit consists of the singly cationic assembly [Cr(C_15_H_9_O_4_)(C_12_H_8_N_2_)(H_2_O)(NO_3_)]^+^ of the Cr(III) coordination complex and one nitrate counter ion, further supplemented by one lattice water molecule (Figure 2). The chrysin ligand is again singly deprotonated at the same phenolic terminal. The deprotonated flavonoid binds the metal ion through that terminal and the keto oxygen atom, thereby generating as previously stated a six-membered metallacyclic ring. Analogous to the bipy ligand fashion in **1**, the phenanthroline chelator binds the metal center through the sp^2^ nitrogen anchors, further stabilizing the complex through another five-membered metallacyclic ring. The oxygen atom of one nitrato ligand binds Cr(III) in a monodentate fashion, with a water molecule also binding the metal ion at the diametrically opposed position. Therefore, the overall coordination geometry around Cr(III) is again distorted octahedral. The two oxygen atoms of chrysin and the two nitrogen atoms of the phenanthroline ligand occupy the equatorial plane of the octahedron, with the oxygen atoms from the nitrato and aqua ligands occupying the axial positions.

The interatomic distances of all oxygen and nitrogen atoms coming from the flavonoids, nitrogenous aromatic bases, nitrato ligands, and the aqua moieties extend to the boundaries of the coordination sphere around Cr(III), with Cr–O and Cr–N distances in the range 1.8911(14)–1.9957(18) Å and 2.0528(18)–2.0838(17) Å, respectively. All of these bond distances and angles around the central metal ion are similar to those encountered in other Cr(III) metal ionic complexes of similar composition.

The nitrato and aqua ligands as well as the lattice counter ions and solvate molecules, together with the deprotonated chrysin ligands, formulate an environment conducive to hydrogen-bonding interactions, involving all these species, thereby extending a network of interactions, collectively stabilizing both arising lattices (Table S1).

### 2.3. FT-IR Spectroscopy

The FT-IR spectra of compounds **1** and **2**, in KBr, signify the coordination of chr and aromatic chelators (bipy and phen) to the Cr(III) metal ion. Specifically, there is initially a distinct change in the zone of stretching vibrations of the O–H bonds relative to that of free chrysin. These vibrations occur at lower frequencies, with more distinct peaks at 3368 and 3203 cm^−1^ for **1** (Figure S1A) and at 3391 and 3067 cm^−1^ for **2** (Figure S1B). They are characterized by low intensities. The reduction in the intensity of these peaks indicates the loss of a phenyl O–H group during the formation of a coordination bond with the Cr(III) metal ion center. Moreover, the vibrational frequency of C = O shifts to a lower frequency of 1642 and 1635 cm^−1^ for **1** and **2**, respectively, suggesting that Cr(III) binds chrysin via the oxygen atom of the carbonyl group at position 4 of the C flavonoid ring, consistent with previously reported materials synthesized with trivalent metal ions and chrysin [25,26]. Considering the above observations, a weak and a moderate peak in the 400–500 cm^−1^ region, at 521 cm^−1^ and 427 cm^−1^ for **1** and at 521 cm^−1^ and 436 cm^−1^ for **2** (Figure S1A,B), respectively, representing the vibrational frequencies of the Cr–O and Cr–N bonds, confirm Cr(III) binding to both chrysin and aromatic chelators. The described tentative assignments are consistent with infrared frequencies previously attributed to O,N-containing ligands bound to the Cr(III) metal ion [27,28].

### 2.4. UV-Visible Spectroscopy

**Figure 3:**
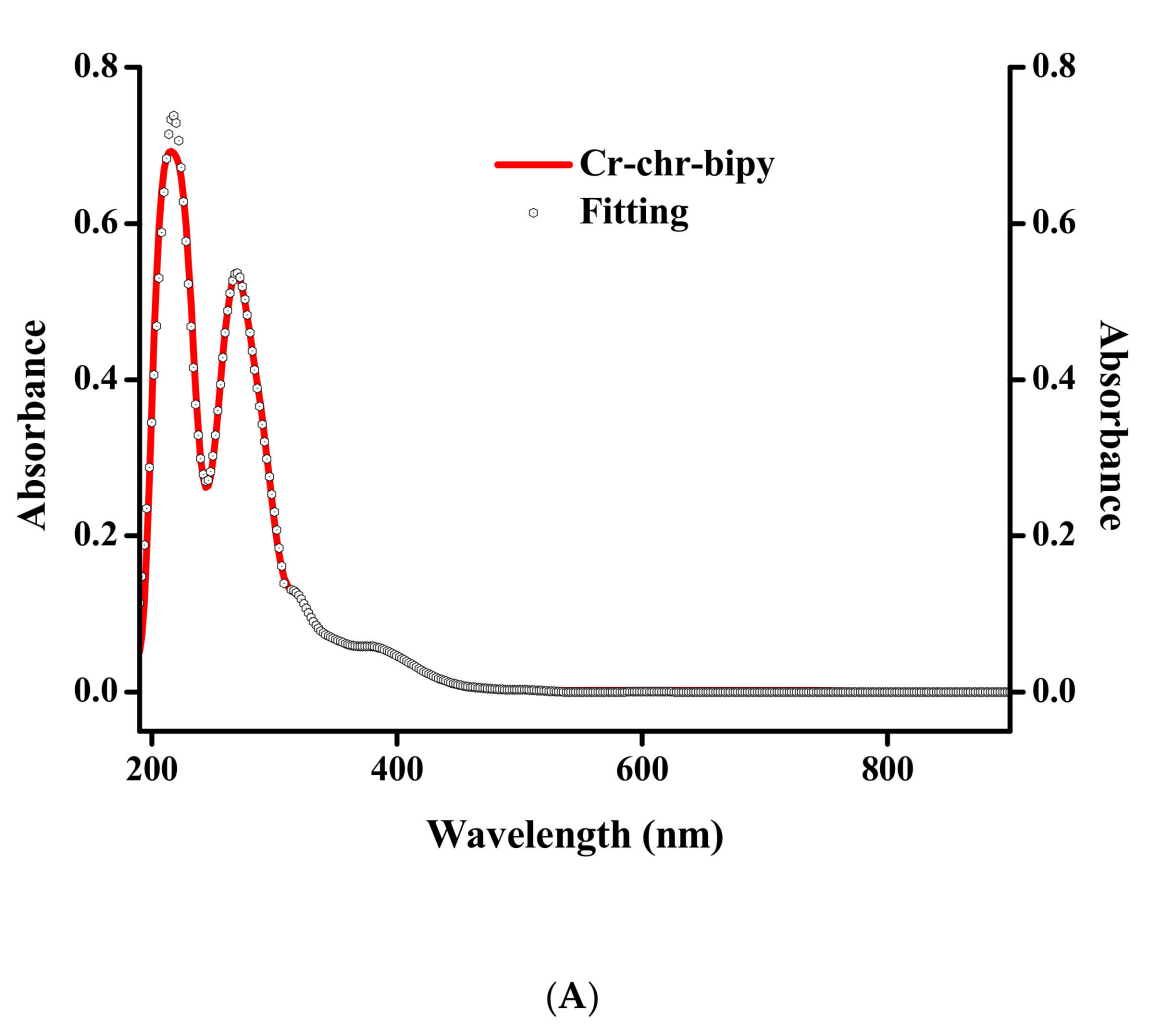

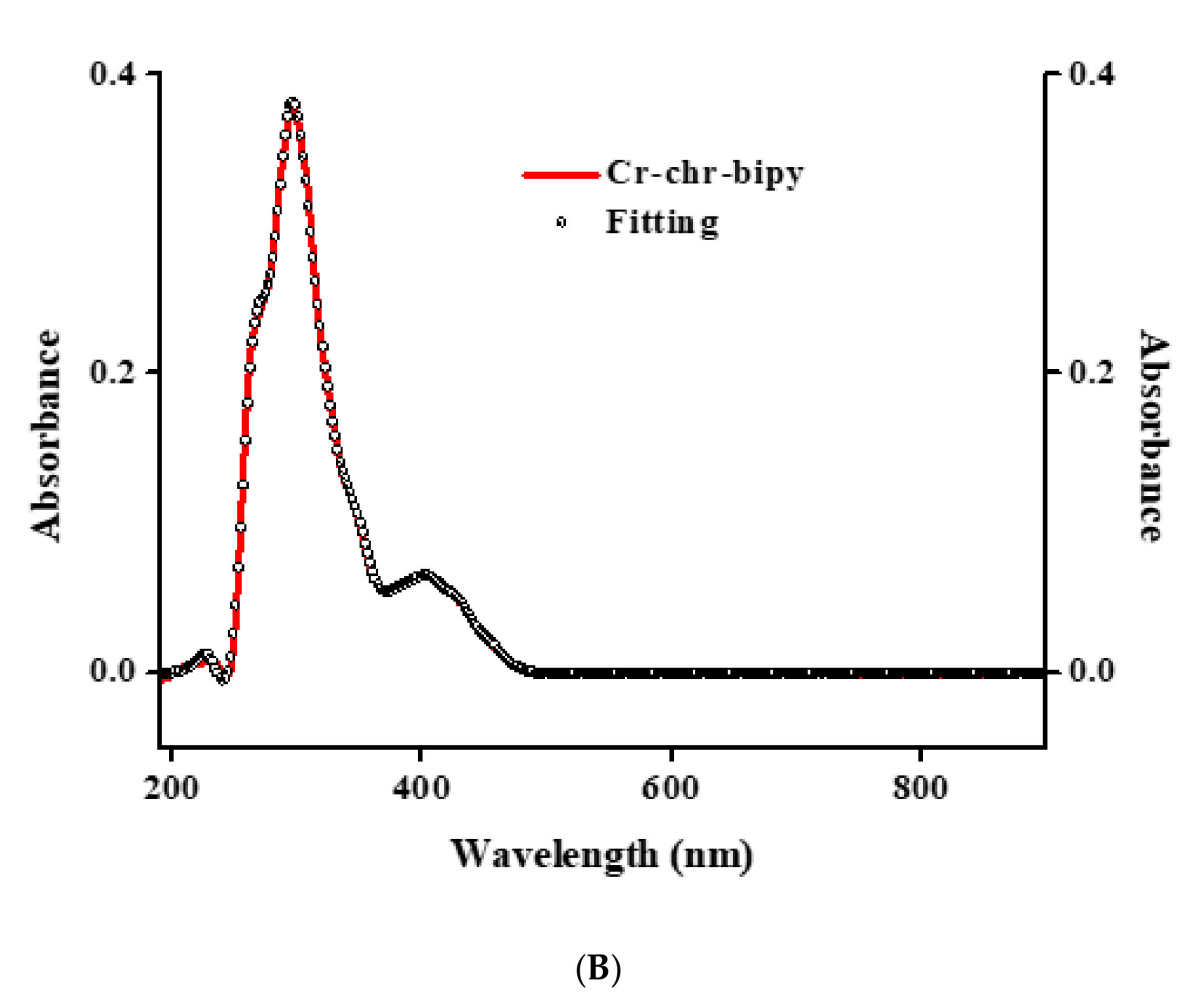
(**A**) UV-Visible spectrum (red line) and spectral fitting (circles) of compound **1** in methanol (10^−5^ M). (**B**) UV-Visible spectrum (red line) and spectral fitting (circles) of compound **1** in DMSO (10^−5^ M).

**Figure 4:**
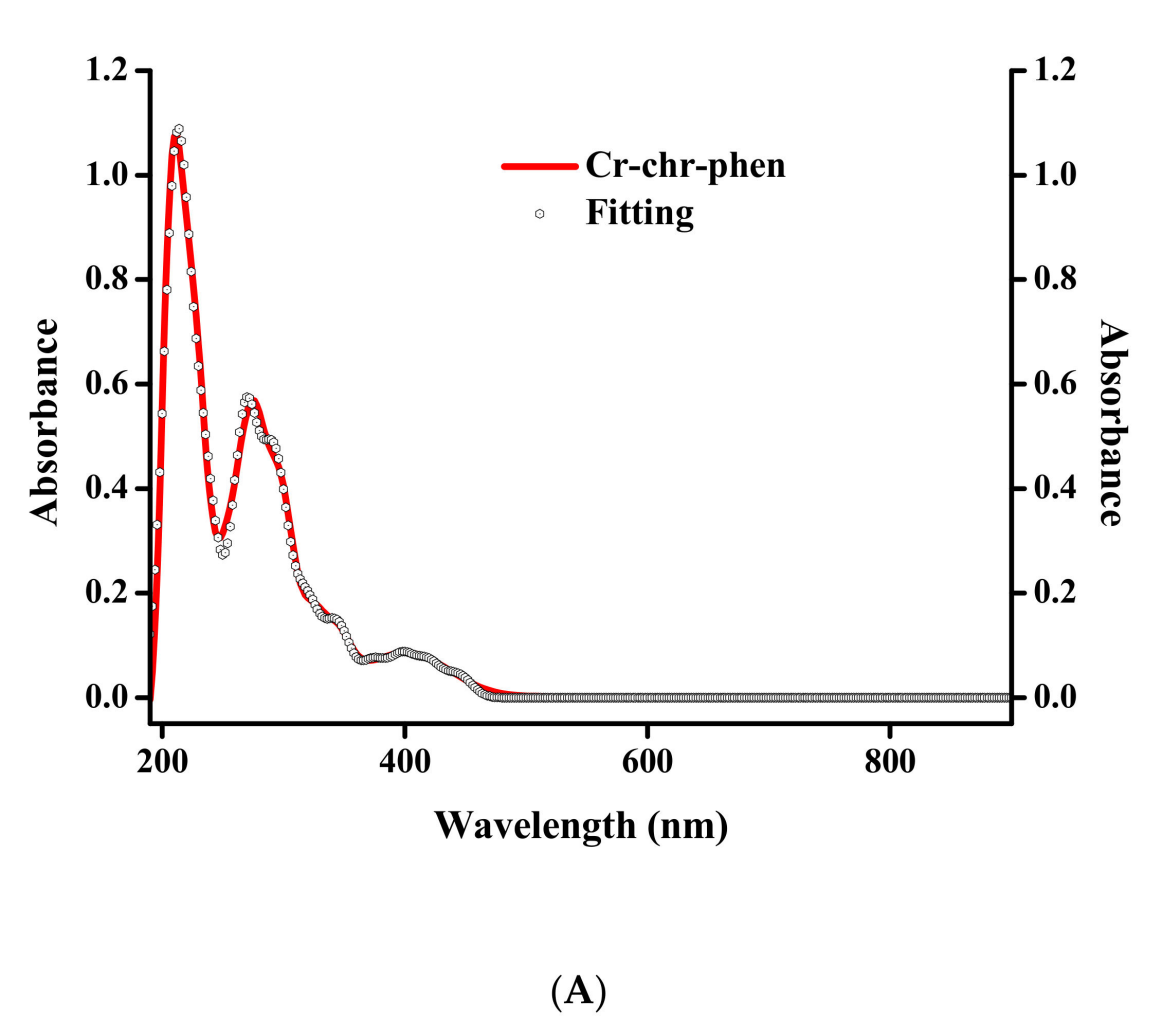

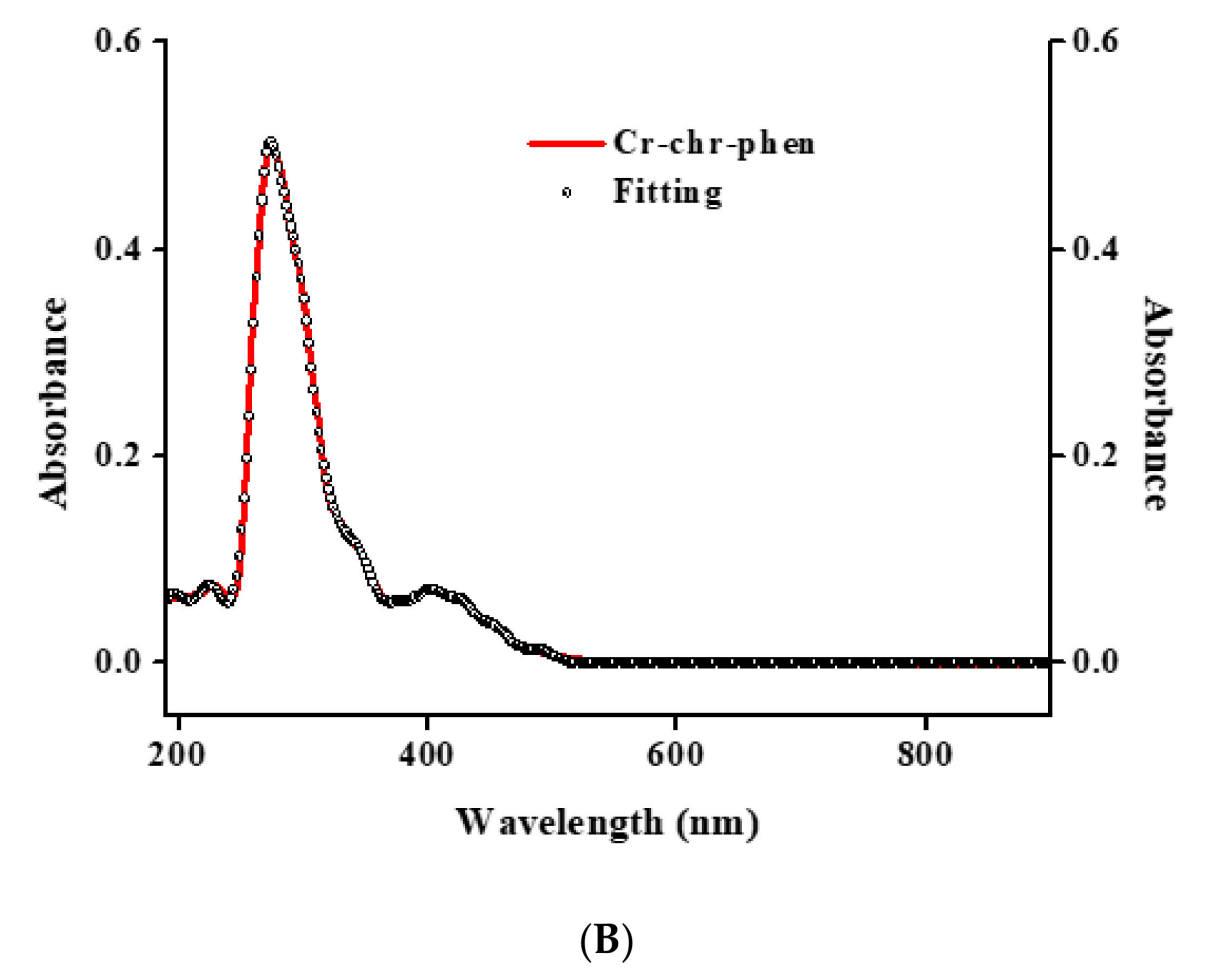
(**A**) UV-Visible spectrum (red line) and spectral fitting (circles) of compound **2** in methanol (10^−5^ M). (**B**) UV-Visible spectrum (red line) and spectral fitting (circles) of compound **2** in DMSO (10^−5^ M).

Electronic spectroscopy measurements were made on both compounds in methanol and DMSO solvents (Figure 3 and Figure 4). The spectra of **1** and **2** in methanol (Figure 3A and Figure 4A) show, at relatively high energies, well-formed feature peaks at 399 and ~341 nm (**1**) and at 400 and ~341 nm (**2**), respectively. At even higher energies, well-defined absorption bands emerge at 293 and 206 nm (**1**) and at 274 and 206 nm (**2**). The absorption features are likely due to d–d transitions, which are typical for a Cr(III) d^3^ octahedral species [26]. The spectra of **1** and **2** in DMSO (Figure 3B and Figure 4B) exhibit a band at 400 nm and a shoulder at ~341 nm (**1**) as well as a band at 402 nm and a shoulder at ~341 nm (**2**), respectively. At higher energies, the spectra show a well-defined absorption band at 298 nm (**1**) and 276 nm (**2**), respectively. From the identifiable features (Table S2), the band around 400 nm could be tentatively attributed to the ^4^A_2g_ → ^4^T_1g_ transition. The shoulder-like feature at 341 nm could be assigned to the chrysin ligand bound to Cr(III), with the band at 293 (**1**) and 274 (**2**) most likely belonging to the bipy and phen ligands bound to Cr(III) in the respective complex assemblies. The features at 206 nm (**1**,**2**) were attributed to charge transfer processes. No further definitive assignments could be made in the absence of detailed specific studies. The spectra of both compounds are different from that of Cr(III)_aq_ in the respective solvent systems [22,29], thus indicating that the coordination sphere of Cr(III) is likely to be retained in solution (vide infra).

### 2.5. ESI-MS Spectrometry

The ESI-MS spectra of compounds **1** and **2** ware recorded in methanol in positive mode. The spectrum of compound **1** exhibits the following patterns: M_1_1_ = [M/2-2H_2_O-H+CH_3_OH]^+^, m/z = 492.0771-495.0800 (z = 1) and M_1_2_ = [M/2-2H_2_O-H]^+^, m/z = 460.0512-461.5544 (z = 1), as shown in Figure S2. The spectrum of compound **2** exhibits a pattern of three peaks, depicted as M_2_1_ = [M-H_2_O-OH]^+^, m/z = 530.0541-533.0624 (z = 1), M_2_2_ = [M-NO_3_-H_2_O-H+CH_3_OH]^+^, m/z = 516.0742-519.0640 (z = 1), and M_2_3_ = [M-NO_3_-H_2_O]^+^, m/z = 485.0353-488.0726 (z = 1), as shown in Figure S3. The molecular ions M_1_1_ and M_2_1_ reflect the basic peak of the spectra rising to 100% of the relative intensity of all spectral components.

### 2.6. Luminescence Studies

**Figure 5:**
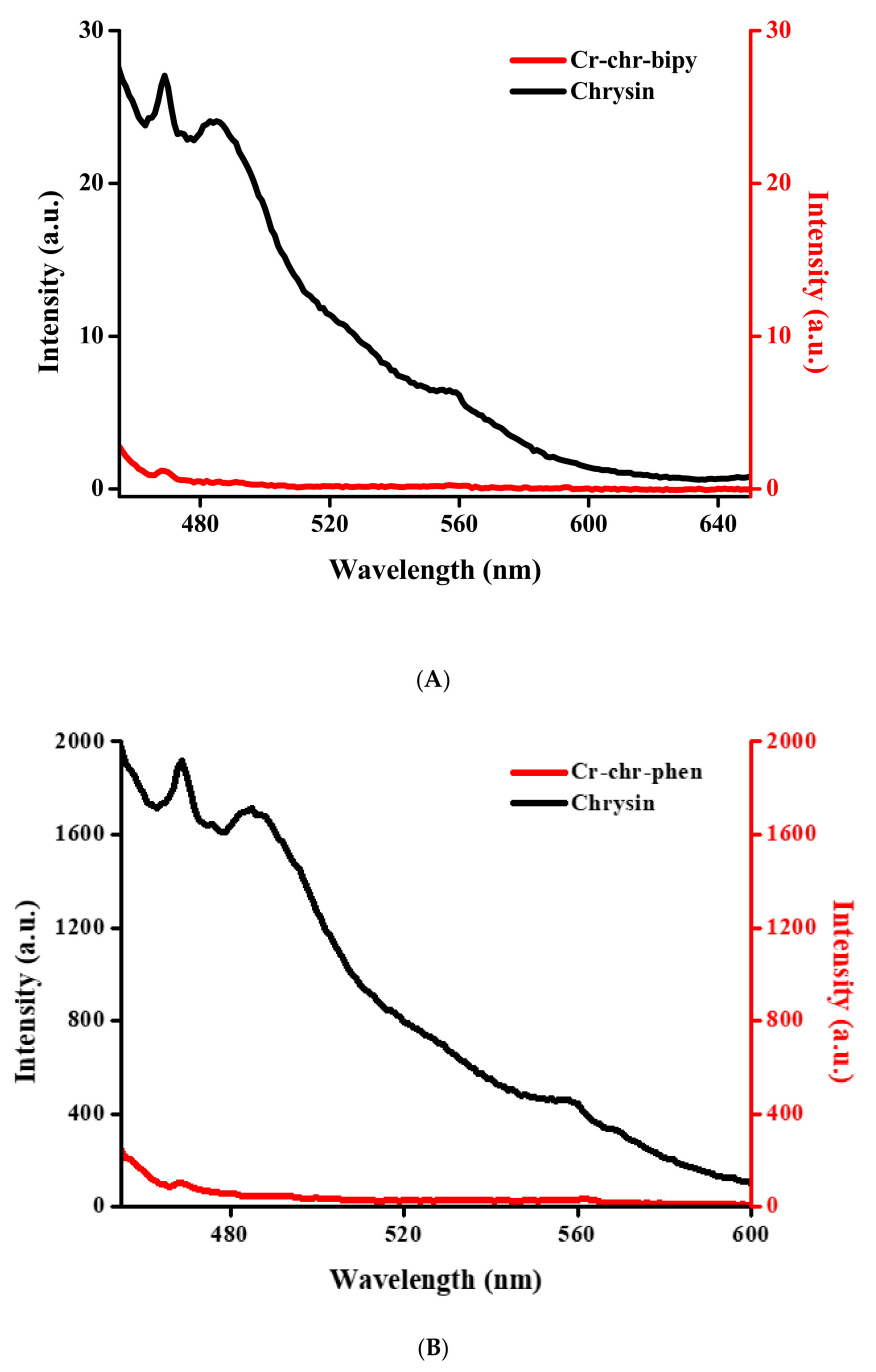
(**A**) Luminescence spectrum of compound **1** compared to that of the free chrysin ligand in solid state. (**B**) Luminescence spectrum of compound **2** compared to that of the free chrysin ligand in the solid state.

The luminescence properties of compounds **1** and **2** as well as that of pure free flavonoid ligand and aromatic chelators (bipy and phen) were studied in the solid-state at 25 °C. The spectral data reveal that both compounds exhibit a strong quenching effect related to the chrysin ligand at 469 (λ_ex_ = 445) for **1** and 468 (λ_ex_ = 446) for **2**, as shown in Figure 5A,B. With respect to the aromatic chelator bound to Cr(III), there is no feature corresponding to that of the ligand.

### 2.7. Cell Survival Results

#### 2.7.1. Toxicity in C2C12 Myoblasts in 24 and 48 h

**Figure 6:**
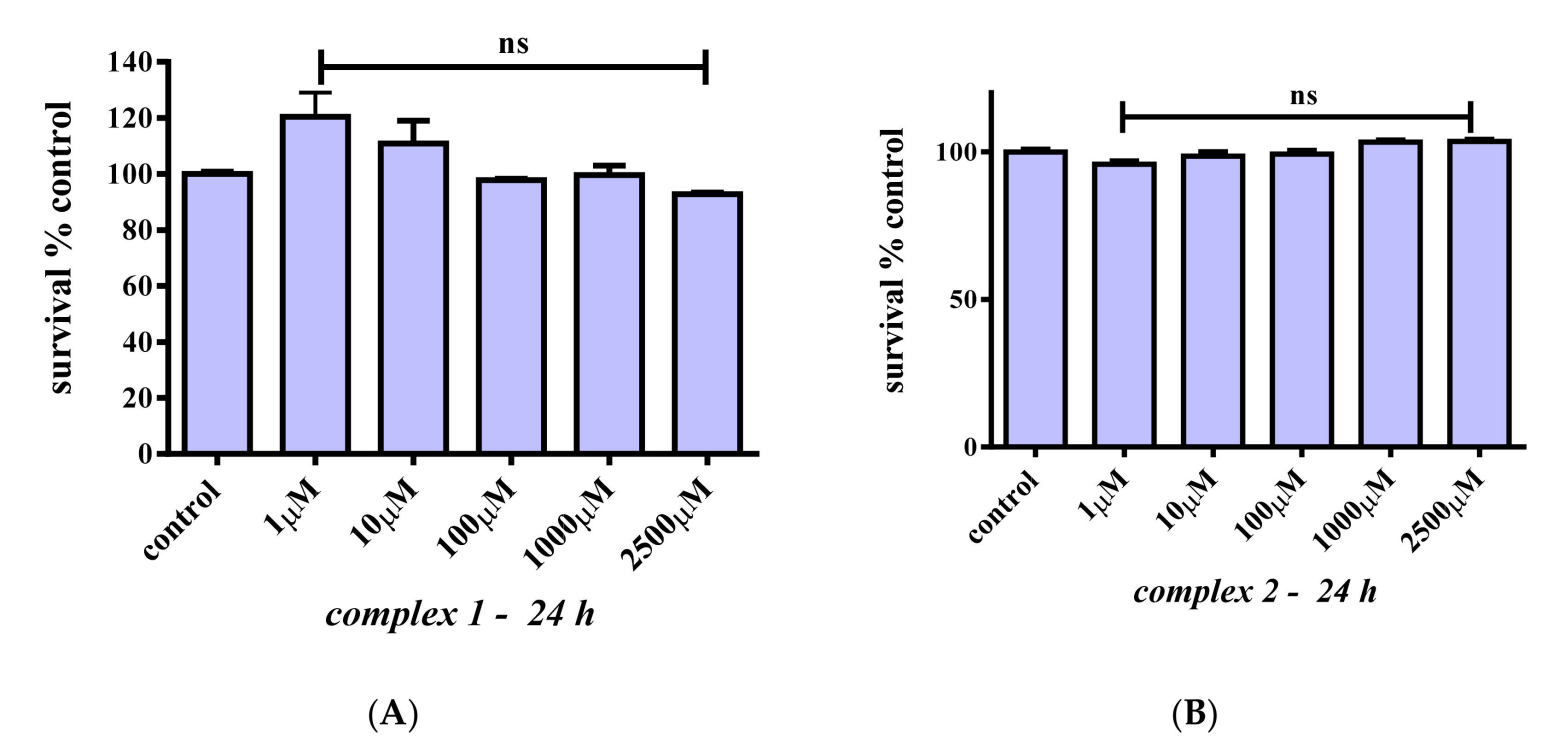
Percent change of cell survival in C2C12 myoblasts, following treatment at various concentrations (1–2500 μM) of **1** (**A**) and **2** (**B**) for 24 h. Values represent the mean value of several (n = 3) independent experiments. Vertical bars represent SEMs. ns: not statistically significant (*p* > 0.05).

**Figure 7:**
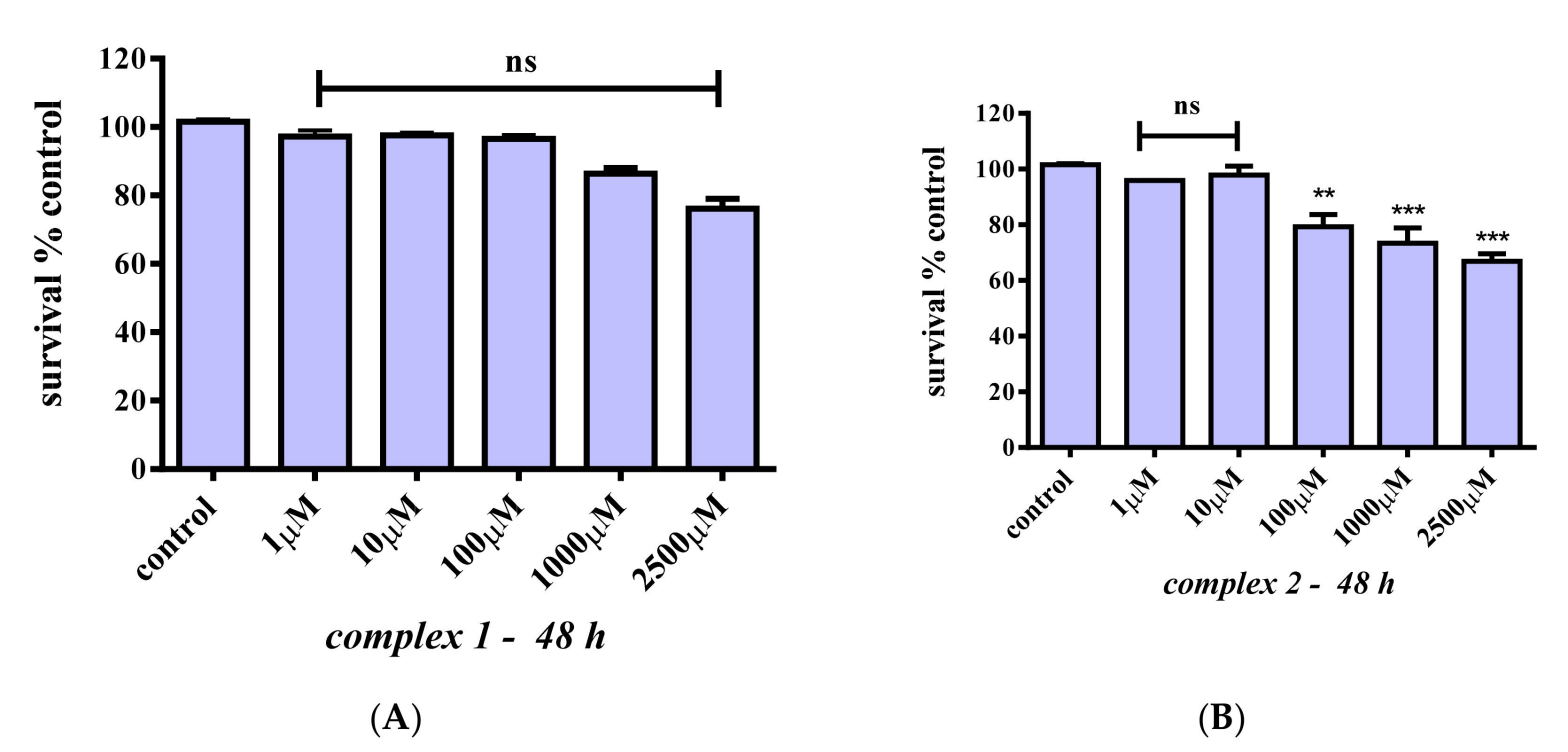
Percent change of cell survival in C2C12 myoblasts, following treatment at various concentrations (1–2500 μM) of **1** (**A**) and **2** (**B**) for 48 h. Values represent the mean value of several (n = 3) independent experiments. Vertical bars represent SEMs. **: *p* < 0.01; ***: *p* < 0.001 vs. control. ns: not statistically significant (*p* > 0.05).

To examine whether **1** and **2** affect cell survival rates, C2C12 myoblasts were treated with 1, 10, 100, 1000, and 2500 μM concentrations of the title compounds for 24 h. Figure 6A shows that **1** does not reduce cell survival in the concentration range tested (*p* > 0.05) compared to the control. A slight proliferative, albeit not statistically significant, effect may arise at low concentrations. By the same token, compound **2** does not affect cell survival at any of the concentrations tested (Figure 6B). Given that chromium in its trivalent state has been proven to be kinetically inert, cell survival was also investigated over longer incubation times. Figure 7A shows that **1** does not affect cell survival at low concentrations (1–100 μM), whereas a trend for a concentration-dependent reduction in cell survival is observed for concentrations in the range 1000–2500 μM (albeit not statistically significant). Therefore, **1** does not seem to be toxic at any concentration tested, and a slight reduction in cell survival is observed at 1000 and 2500 μM that amounts to 86.4% and 76.1%, respectively (*p* > 0.05). In the case of compound **2** (Figure 7B), there is no reduction in cell survival at concentrations in the range 1–10 μM, with cell survival rates declining progressively to 79.2% (*p* < 0.01), 73.4% (*p* < 0.001), and 66.9% (*p* < 0.001) for 100, 1000, and 2500 μM, respectively.

#### 2.7.2. Toxicity in C2C12 Mature Myotubes for 24 h

**Figure 8:**
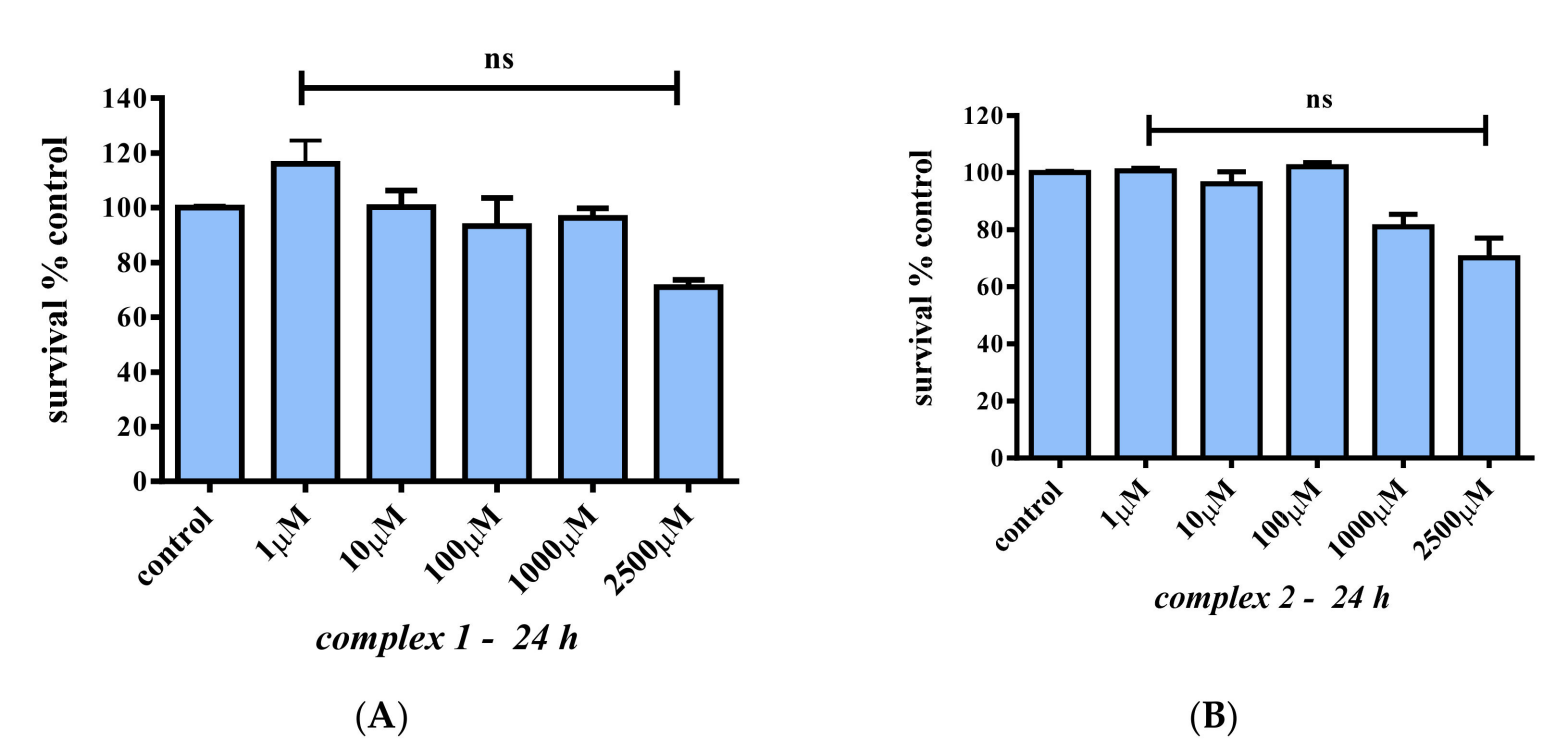
Percent change of cell survival in C2C12 myotubes, following treatment with various concentrations (1–2500 μM) of **1** (**A**) and **2** (**B**) for 24 h. Values represent the mean value of several (n = 3) independent experiments. Vertical bars represent SEMs. ns: not statistically significant (*p* > 0.05).

In an effort to assess the potential cytotoxic effects of **1** and **2** in mature myotubes, differentiated C2C12 (differentiated as described above) were treated with 1, 10, 100, 1000, and 2500 μM concentrations of either **1** or **2**. The results, as shown in Figure 8, indicate that both **1** and **2** do not affect cell survival even at high concentrations (100 μM) compared to the control (*p* > 0.05), whereas both complexes slightly reduce cell survival only at concentrations higher than 1000 μM (70.1% and 71.0%, respectively (*p* > 0.05)).

#### 2.7.3. Oxidative Stress Studies

**Figure 9:**
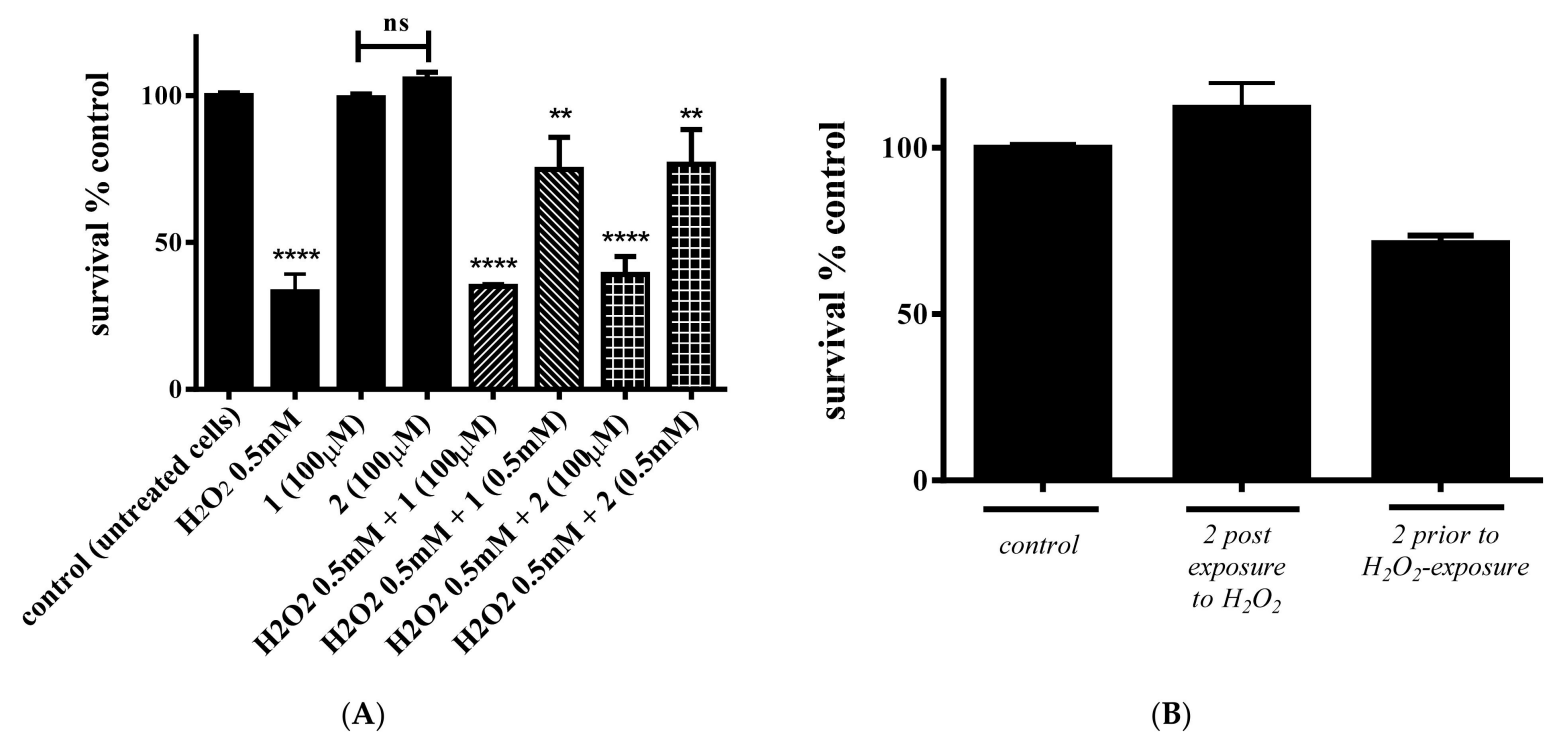
Percent change of cell survival in C2C12 myoblasts, following (**A**) concurrent treatment of cells with either **1** or **2** and H_2_O_2_, and (**B**) treatment with **2** either prior to or following exposure to H_2_O_2_. Values represent the mean value of several (n = 3) independent experiments. Vertical bars represent SEMs. **: *p* < 0.01; **** *p* ≤ 0.0001 vs. control. ns: not statistically significant (*p* > 0.05).

Having investigated the cytotoxicity of **1** and **2**, their potential antioxidant capacity was examined next. For that purpose, cell survival was monitored after exposing cells to H_2_O_2_, which is an agent known to induce ROS production. Experimental conditions were divided in three distinct groups of exposure. First, H_2_O_2_ was added to the cells simultaneously with either **1** or **2** in an effort to assess the cytoprotective effect, when cells are directly exposed to H_2_O_2_. Both compounds were added in 1:1 and 1:5 stoichiometric ratios with H_2_O_2_. More specifically, the selected concentrations were 0.5 mM H_2_O_2_ vs. 100 μM **1** or **2** and 0.5 mM H_2_O_2_ vs. 0.5 mM **1** or **2**. Cells exposed only to H_2_O_2_ were considered as a negative control of the assay. As shown in Figure 9A, cell survival was reduced by ~67% (*p* ≤ 0.0001) when cells are treated with 0.5 mM H_2_O_2_ for 24 h. In the case of **1**, when cells were exposed to H_2_O_2_ and **1** (H_2_O_2_:**1** = 5:1), cell survival amounted to 35.1% (*p* ≤ 0.0001), whereas in the case of **2**, for the same stoichiometric ratio, cell survival amounted to 39.1% (*p* ≤ 0.0001). On the other hand, when cells were exposed to H_2_O_2_ with a 1:1 stoichiometric ratio for both compounds, cell survival amounted to 74.8% and 76.6%, respectively (*p* < 0.01).

In the ensuing experiments, **2** was selected to investigate the potential cytoprotective efficiency when cells are either exposed to H_2_O_2_ and then **2** is added or when **2** is added prior to H_2_O_2_ exposure. In both cases, pretreatment with either **2** or H_2_O_2_ was performed for 2.5 h. As shown in Figure 9B, in the case of H_2_O_2_ pre-exposure, cell survival following the addition of **2** is ~100.0%, indicating the full protection of cells toward ROSs. On the other hand, when cells are pre-treated with **2**, the addition of H_2_O_2_ reduced cell survival by ~29%.

## 3. Discussion

### 3.1. The Premise of Flavonoid Availability in Cell Physiology

Having introduced a basis for the justification of the natural product chemistry giving rise to a variety of flavonoids, it seemed rational to try to use flavonoids in nutrition, health, and disease administration, in bioavailable forms, ensuring bioactivities linked to the composition of the basic core structure, which includes the A, B, and C rings. In line with the notion of the enhancement of flavonoid bioactivity, the herein adopted approach targeted specifically structured flavonoids, thereby generating soluble and bioavailable metal organic complexes. It is in this respect that chrysin was employed in the chemistry with Cr(III) and N,N′-aromatic chelators in the present synthetic and biological study, thus providing insight into natural flavonoids gaining bioavailability and through it being capable of inducing defined biological action(s) supporting cell physiology or quelling pathological aberrations [30]. Collectively, coordination of chrysin to metal ions, such as trivalent Cr(III) linked to diabetes physiology, has globally formulated the scope of the work and provided the impetus to pursue successfully the chemistry presented herein, ultimately leading to the crystallographically characterized complexes **1** and **2** (vide infra).

### 3.2. The Structural Speciation in Ternary Cr(III) Systems Containing Chrysin

Having established the chemical identity of the flavonoids as metal ion binders and seeking to enhance their solubility-bioavailability and hence antioxidant capacity in biologically relevant media, the coordination chemistry of chrysin was pursued with the biogenically relevant metal ion Cr(III) in the presence of ternary N,N′-aromatic chelators. Bearing in mind that the chemically reactive groups on the basic core structure of chrysin include the phenol moieties (C5) and the keto group (C4), we opted for the coordination chemistry with a trivalent metal ion, such as Cr(III). To pursue coordination with a 3d^3^ kinetically inert metal ion, its chemistry should be dictated by factors that might eventually lead to stable species. The incorporation of bipy and phen into the synthetic efforts, as strong N,N′-aromatic chelators acting through their sp^2^ nitrogen anchors, led to new ternary Cr(III)-chr-bipy and Cr(III)-chr-phen systems in methanolic media. The finally isolated materials were crystalline in nature and positively identified through elemental analysis, FT-IR, and X-ray diffraction. The three-dimensional structure of the title compounds reveals cationic metal ionic assemblies, with the central metal ion bound to both the singly deprotonated chrysin and the N,N′-aromatic chelators configured into a distorted octahedral environment bearing two axially poised aqua (for **1**) as well as one aqua and one monodentate nitrato ligand (**2**). Worthy of note are the facts that (a) the chrysin flavonoid is deprotonated at the phenolic moiety abutting the keto group, thus supporting the formation of a stable six-membered metallacyclic ring sustained by the A-C ring part of the flavonoid core; (b) further contribution to the stability of the ternary assembly is provided by the bipy and phen chelators, which are poised into the equatorial plane of the octahedron and form another nitrogen-containing five-membered metallacyclic ring; (c) the nitrato group (in **2**) in the axial position is monodentate, thus signifying its presence in the starting reagent of the reaction mixture leading to both compounds **1** and **2**, yet binding to Cr(III) only in **2**; (d) the presence of the coordinated aqua ligands in both species emphasizes the importance of a good leaving group bound to a metal ion and, for that matter, to a kinetically inert one. The potential of the aqua leaving group being the agent, through which the complex and thus the metal ion can interact with biologically important targets in cellular media is quite evident and distinct in the biology of organisms. All of the above properties in the crystallographically characterized metal-organic assemblies are different from those reported in the literature (most of them synthesized in our lab) based on M(II) and M(III) assemblies. Reported Zn(II) complexes of ternary systems include [Zn(CH_3_COO)(C_15_H_9_O_4_)(C_10_H_8_N_2_)]_9_·CH_3_OH and [Zn(C_15_H_9_O_7_)(C_10_H_8_N_2_)Cl]·(CH_3_OH)·1.5H_2_O, containing chrysin and quercetin, respectively, and the auxiliary N,N′-phen aromatic chelator. Juxtaposed to those stand the complex assemblies [Zn_2_(L)_2_(phen)_2_(MeOH)_2_]^2+^ (L = morin) as well as the Cu(II) complex [Cu_2_(L)_2_(phen)_2_](NO_3_)_2_·MeOH (L = chrysin), with both of them being dinuclear assemblies [31,32,33]. Other complex assemblies include Ga(III)-chrysin [Ga(C_15_H_9_O_4_)(C_12_H_8_N_2_)(NO_3_)(CH_3_OH)]^+^ containing phen, [Ga(C_15_H_9_O_4_)(C_10_H_8_N_2_)_2_]^2+^ containing bipy, and [Ga(C_15_H_9_O_4_)_2_(C_3_H_4_N_2_)_2_]^+^ containing imidazole [26], all of them being mononuclear assemblies such as the ones described in this work. A binary complex of morin flavonoid with Fe(III) [34] bearing the molecular structure [Fe(morin)_3_] has also been reported in the literature. The structures of the herein reported compounds **1** and **2** are different (in coordination geometry, coordination sphere composition, etc.) from the ones cited above from the point of view of the metal ion employed and the nature of the flavonoid bound to the metal ion’s coordination sphere, thus showing the distinct effect of metal ion chemistry on the coordination of flavonoid molecules. The overall reported results exemplify the diversity of chelation of flavonoid molecules, based on their own structure, to different metal ions M (II,III), thereby presaging the diversity of such structures in biological media extending across the hierarchy of species in the metallobiology of (patho)physiology of organisms.

Collectively, both crystallographically characterized materials **1** and **2** synthesized and isolated in the course of this work exemplify the usefulness of structural speciation in systems to be employed in biological investigations. Their completed physicochemical characterization in the solid state and in solution provide ample grounds for their employment in further biological experiments and/or evaluation of their role in biological processes of importance to cellular (patho)physiology (vide infra). Given the unique nature of the complex assemblies in the present work and the global picture of their role(s) in (patho)physiological biology, their unraveled biochemical profile is quite distinct in providing information about the system for which that was called upon to investigate. To that end, a new challenge arises steadily into the growing family of species synthesized and crystallographically characterized in bioinorganic chemistry: that of inquiring into the roles of metal-organic species, containing flavonoids, in the biology of organisms either under physiological conditions or under aberrant states, such as that of oxidative stress. That work lies ahead and promises new information on metal-flavonoid interactions and their correlation with structural composition reflecting biological activity and potency in physiology and disease.

### 3.3. The Antioxidant Potency

Development and employment of advanced materials in metallopharmaceutical applications relies heavily on full characterization of the overall (bio)chemical profile of any potential such compound-factor. This effort, in turn, relies on (a) structural and physicochemical data and (b) biological data emanating from (a)toxicity and biomimetic/bioactivity tests. In the present study, the title compound species **1** and **2** were employed to investigate their potential as antioxidants in pathologies related to oxidative stress. Prior to that, their cytotoxic profile was investigated, since low or zero toxicity is of fundamental significance for further consideration. In so doing, both **1** and **2** were used for the treatment of C2C12 myoblasts and mature myotubes. Both complex compounds appear to be non-toxic, even at high concentrations (1000 μM), for both short (24 h) and longer times (48 h) of incubation.

In view of the aforementioned grounds, oxidative stress stands as a “negative state,” related to the pathogenesis of various diseases [35]. To that end, an alternative approach to the treatment of skeletal myopathies, brought about by oxidative stress as a result of aberrant mitochondrial metabolism (excess of free radicals), could be the employment of drugs and bioactive substances that alleviate oxidative stress produced by mitochondrial dysfunction or any other source [36].

There is a plethora of antioxidants that can be used to decrease the amount of reactive oxygen species (ROSs) in cells exposed to conditions of oxidative stress. The efficiency of bioactive substances, however, may vary mainly due to bioavailability issues. For instance, several flavonoids are insoluble in water and hence cannot reach biological targets to exert their activity. Metal ion complexation has been proven to overcome such obstacles. In the present study, Cr(III) complexation of a specific flavonoid (i.e. chrysin) took place in an effort to increase solubility and enhance the prospect of employment of such compounds in biological processes.

The present study targets the in vitro characterization of the effects of ROSs on C2C12 cells and assesses the efficacy of antioxidant treatment in alleviating these toxic effects. The monitoring of this effect was pursued through cell survival screening, since cell death is the main phenotypic event of ROSs in cells. Elevated concentrations of H_2_O_2_ exposure resulted in increased intracellular ROS levels and decreased cell metabolic activity.

In the framework of such a research effort, the potential antioxidant effects of Cr(III) complexes with flavonoids, known for their antioxidant capacity in C2C12 mouse myoblasts, were investigated. The specific cell line is a common biological model in the lab and has been selected due to the properties it ferries into research pertaining to the differentiation of myoblasts [37], osteoblasts, myogenesis, and the potential exploration of mechanistic biochemical pathways in (patho)physiological states. A significant collection of such pathological states involves and/or includes oxidative stress. Under oxidative stress caused by 0.5 mM H_2_O_2_, the addition of **1** and **2** at certain defined stoichiometric ratios ameliorated the extent of cell death induced by the high levels of ROSs. Consequently, the results provide insight into the potential application of **1** and **2** toward alleviation of oxidative damage to muscles. Moreover, the highest cytoprotective activity was shown when **1** and **2** were added following H_2_O_2_ exposure.

Moreover, worth noting is the fact that several Cr(III) complexes have been shown to induce ROSs (e.g., Cr(III)-picolinate) that result in decreased cell survival and proliferation. That does not hold for all complex species of Cr(III) bound to organic substrates, as there are complexes that do not act in a similar fashion [22]. That is the case of the Cr(III)-citrate complex, indicating that the nature of substrates to which Cr(III) is bound does emerge as crucial to the biological activity it exemplifies.

### 3.4. Mechanistic Considerations

Studies in the past have established the antioxidant capacity of flavonoids as a result of their structural composition and the existence of selected -OH groups located in key parts of all rings in their molecular skeleton. The herein selected flavonoid, chrysin, possesses the basic core structure (absence of a catechol moiety in the 3′,4′ positions of the B ring) thought to play a crucial role in the promotion of antiradical scavenging activity [27,38]. Beyond that attribute, however, the process of metal ion complexation [39,40] enhances that antiradical and hence antioxidant activity of the flavonoid, as this has been attested to through the existing literature, employing actually the Cr(III) ion as the metal center [41]. For the first time, the herein described work exemplified the interactions involved between Cr(III) and the flavonoid chrysin, presenting structurally well-characterized complex forms that did not exist in the past, with chrysin bound to Cr(III) in an octahedral environment formulated through the chelation of yet another aromatic N,N′-binder (phen and bipy). Given that such a coordination environment is conducive to antioxidant activity, as has been shown in vitro in C2C12 myoblast cultures, potential sites through which the exemplified activity was observed could include the remaining free 7-OH moiety on the A ring, and the water and nitrato moieties could serve as sites of biochemical reactivity with reactive oxygen species generated in human biological fluids through metal-linked or nonmetal-linked processes arising as a result of exerted pressure through oxidative stress [42]. To that end, the process of complexation of chrysin to Cr(III) (a) enhances its solubility in biological fluids, thus rendering it more bioavailable to sequestering reactive oxygen species when and where the latter are generated, (b) enhances the emerging antioxidant activity at an oxidatively burdened site in a cell, when the latter comes under stress, and (c) stands in line with the properties emanating through observations on antioxidant activity of Cr(III)-complexed flavonoids [43]. This is undoubtedly an advantage in the antioxidant arsenal of a flavonoid-containing material, seeking competitive inclusion in potential oxidative stress-counteracting medication, especially so since (a) chrysin itself is sparingly soluble in aqueous media, and (b) free Cr(III) ions do not possess solubility-bioavailability attributes around the physiological pH value. Further inquiry into the biology of the well-defined species, presented here through in vitro studies, are expected to shed light onto interactions with (sub)cellular molecular targets, thereby aiding in the development of future efficient metallotherapeutics.

## 4. Experimental

### 4.1. Materials and Methods

All manipulations were carried out under aerobic conditions, using metal ion salts and flavonoid ligands without further purification. Cr(NO_3_)_2_·9H_2_O was purchased from Fluka (Buchs, Germany). Chrysin (chr), 1,10-phenanthroline (phen), and solvents (methanol, diethyl ether, dimethylsulfoxide (DMSO), and n-hexane) were purchased from Sigma-Aldrich (Steinheim am Albuch, Germany). 2,2′-Bipyridine (bipy) was supplied by Panreac (Barcelona, Spain). The water used in the biological experiments was of nanopure quality.

### 4.2. Physical Measurements

FT-Infrared spectra measurements were taken on a Thermo Electron Corp., Madison, WI, USA. FT-Infra Red IR-200 spectrometer, using KBr pellets. UV-Visible measurements were carried out on a Hitachi U-1900 (Hitachi, Tokyo, Japan) spectrophotometer in the range from 190 to 900 nm in methanol and DMSO with a 1200 nm/min scan speed. Spectral fitting of solutions in **1** and **2** in methanol was pursued by using the SYSTAT software Inc. Peakfit program (Version 4.11, Systat Software, Inc., Point Richmond, CA, USA). In the course of such an effort, the use of Savitzky-Golay algorithms includes the following parameters: (a) maximum number of iterations = 8000–10,000; (b) significant digits = 6; (c) a full curvature matrix was used in the fitting process; (d) fit extent = every other point until R^2^ reveals a value of 0.9999 ± 0.0001.

A ThermoFinnigan Flash EA 1112 CHNS elemental analyzer (Thermo Scientific, Waltham, MA, USA) was used for the simultaneous determination of carbon, hydrogen, and nitrogen (%). The analyzer operation is based on the dynamic flash combustion of the sample (at 1800 °C) followed by reduction, trapping, complete GC separation, and detection of the products. The instrument is (a) fully automated and is controlled by a PC via the Eager 300 dedicated software (Thermo Scientific, Waltham, MA, USA), and (b) capable of handling solid, liquid, or gaseous substances.

#### 4.2.1. ESI-MS Spectrometry

Electrospray ionization mass spectrometry (ESI-MS) infusion experiments were carried out on a ThermoFisher Scientific model LTQ Orbitrap Discovery MS (Bremen, Germany). The compounds **1** and **2**, containing the chrysin ligand with the aromatic chelator bipy for **1** and phen for **2**, were dissolved in methanol and introduced into the ESI source of the MS at a flow rate of 5 μL/min, using an integrated syringe pump. The infusion experiments were run using a standard ESI source, operating in a positive ionization mode. Source operating conditions were a 3.7 kV spray voltage and a 320 °C heated capillary temperature.

#### 4.2.2. Photoluminescence

Solid-state emission and excitation spectra (200–900 nm) were recorded on a Hitachi F-7000 fluorescence spectrophotometer from Hitachi High-Technologies Corporation (Tokyo, Japan). The employed split widths (em, ex) were 5.0 nm, and the scan speed was 1200 nm·min^−1^. All measurements were carried out at room temperature. The entire system was supported by the appropriate computer software, FL Solutions 2.1 (Hitachi High-Technologies Corporation, Tokyo, Japan), running on Windows XP.

### 4.3. Materials Preparation

**Synthesis of [Cr(C_15_H_9_O_4_)(C_10_H_8_N_2_)(H_2_O)_2_]_2_(NO_3_)_4_∙3H_2_O** (**1**). A quantity of Cr(NO_3_)_3_ 9H_2_O (0.20 g, 0.50 mmol) was placed in a 50 mL round bottom flask and dissolved in 10 mL of methanol under continuous stirring. Chrysin (Sigma-Aldrich, Germany) (0.13 g, 0.50 mmol) was then added to the blue solution under continuous stirring. Upon addition of chrysin, the color of the solution turned greenish. Subsequently, the resulting solution was heated mildly for approximately 5 min. In the next phase, bipy (Panreac, Spain) (0.080 g, 0.50 mmol) was added gradually under continuous stirring. The resulting heterogeneous mixture turned darker green. The ternary reaction mixture was brought to reflux and kept there for about 4 h. The emerging brownish clear solution was then allowed to return to room temperature. Subsequently, the reaction solution was filtered, followed by the addition of n-hexane through a vapor diffusion method. After a period of few days, prismatic red crystalline materials were collected by filtration and dried in the open air. Yield: 0.18 g (~55%). Anal. Calcd. For **1**, **[Cr(C_15_H_9_O_4_)(C_10_H_8_N_2_)(H_2_O)_2_]_2_(NO_3_)_4_∙3H_2_O** (C_25_H_24_CrN_4_O_13.50_, M_r_ 648.48): C 92.52, H 7.40, N 17.27. Found: C 92.49, H 7.38, N 17.22.

**Synthesis of [Cr(C_15_H_9_O_4_)(C_12_H_8_N_2_)(H_2_O)(NO_3_)](NO_3_)∙H_2_O** (**2**). A quantity of Cr(NO_3_)_3_.9H_2_O (0.20 g, 0.50 mmol) was placed in a 50 mL round bottom flask and dissolved in 10 mL of methanol under continuous stirring. Chrysin (0.13 g, 0.50 mmol) was then added to the blue solution under continuous stirring. Upon addition of chrysin, the color of the solution turned greenish. Subsequently, the resulting reaction mixture was heated mildly for approximately 5 min. In the next step, phenanthroline (Sigma-Aldrich, Germany) (0.090 g, 0.50 mmol) was added gradually under continuous stirring. The resulting heterogeneous mixture turned darker green. The ternary reaction mixture was refluxed for 4 h until the reaction mixture turned dark brownish. The clear solution was then allowed to return to room temperature and, following filtration, a slow addition of diethyl ether through a vapor diffusion method was pursued. After a period of a few days, prismatic green crystalline material was collected through filtration and dried in the open air. Yield: 0.21 g (~65%). Anal. Calcd. For **2**, **[Cr(C_15_H_9_O_4_)(C_12_H_8_N_2_)(H_2_O)(NO_3_)](NO_3_)∙H_2_O** (C_27_H_21_CrN_4_O_12_, M_r_ 645.48): C 50.20, H 3.25, N 8.68. Found: C 50.17, H 3.21, N 8.64.

### 4.4. X-ray Crystal Structure Determination

X-ray quality crystals of the compounds **1** and **2** were grown from a mixture of methanol-diethyl ether. Important crystallographic data for the studied samples are listed in Table 2. Crystals of **1** and **2** were taken from the mother liquor and mounted at room temperature on a Bruker Kappa APEX II diffractometer (Bruker, Germany) equipped with a triumph monochromator, using Mo *K* radiation. Unit cell dimensions were determined and refined by using the angular settings of at least 192 high intensity reflections (>6σ(Ι)) in the range 19 < 2 < 40°. Intensity data were recorded using and scans. The frames collected were integrated with the Bruker SAINT software package (Bruker, Madison, WI, USA) [44] using a narrow-frame algorithm. Data were corrected for absorption using the numerical method (SADABS) based on crystal dimensions [45]. Data refinement (full-matrix least-squares methods on *F*^2^) and all subsequent calculations were performed using the Crystals version 14.61_build_6236 program package (University of Oxford, Oxford, UK) [46]. The structures were solved through the SUPERFLIP method [47]. Molecular illustrations were drawn using the Diamond 3.2e2 crystallographic package (Bonn, Germany) [48]. All non-hydrogen atoms were refined anisotropically except for the disordered atoms from the nitrate counter anions and the solvate water molecules. All of the latter atoms were refined isotropically with fixed occupancy factors, previously determined when refining with fixed isotropic factors of 0.05.

### 4.5. Biological Studies

#### 4.5.1. Cell Culture

In the present study, the C2C12 cell line (mouse myoblasts) was employed to investigate the biological profile of the title chromium compounds toward cell toxicity and antioxidant activity. Cells (both myoblasts and differentiated myotubes) were cultured in 75 cm^2^ cell culture flasks, under appropriately chosen conditions (5% CO_2_ at 37 °C and standard humidity), in Dulbecco’s modified Eagle’s medium (DMEM) (Sigma, Steinheim, Germany). Culture media were supplemented with 10% fetal bovine serum (FBS) (Biochrom, Berlin, Germany) and 1% penicillin-streptomycin (Biochrom, Berlin, Germany) prior to use. All experiments were run at least in triplicate, employing cells with a low passage number (P3–P6). Induction of cell differentiation (myotube formation) was performed after cell density had reached ≃70% (Day 0). Cells were then exposed to a culture medium supplemented with donor horse serum (Biowest) at a content of 2% *v/v*. A differentiation medium was replaced every two days until the formation of myotubes (10 days).

#### 4.5.2. Cell Viability and Proliferation Studies

Cell viability rates were determined by the XTT (sodium 3-[1-(phenylaminocarbonyl)-3,4-tetrazolium]-bis(4-methoxy-6-nitro)benzenesulfonic acid hydrate) (Cell Signaling) dye reduction assay. In brief, cells (5000 cells/well) were seeded in 100 μL in 96 well-plates and incubated overnight. Cells were then treated with the title compounds at several concentrations (1 μM–2.5 mM) for 24 h. The XTT detection solution was prepared according to manufacturer instructions (electron coupling solution to XTT Reagent (1:50 volume ratio)). A volume of 50 μL of XTT was then added to each well, and the culture plate was incubated for four additional hours. Subsequently, the absorbance was measured using a microplate spectrophotometer at 450 nm, as described elsewhere [49,50]. According to the employed procedure, the tetrazolium salt XTT is reduced to a highly colored formazan dye by dehydrogenase enzymes in metabolically active cells. This conversion occurs only in viable cells, with the amount of formazan produced being proportional to the viable cells in the sample.

Fresh stock solutions of the title compounds **1** and **2** were prepared in DMEM (1% penicillin-streptomycin, 10% FBS). Prior to any experimental procedure, the solubility of the employed compound in water was assessed. All solutions were freshly prepared prior to all experiments (2.5 mM initial stock solution), followed by sterile filtration. Final working concentrations were added directly to the cell cultures and incubated for the desired time periods according to the protocols followed.

#### 4.5.3. Oxidative Stress Studies

To investigate the potential protection of the title compounds from reactive oxygen species (ROSs), cells were incubated in the presence of H_2_O_2_ (a known, well-established redox agent). Cr(III) complexes were added (a) prior to H_2_O_2_ exposure, (b) following H_2_O_2_ exposure, and (c) simultaneously with H_2_O_2_. The treatment protocol is as follows: the first group was treated with H_2_O_2_ for 2.5 h and then either **1** or **2** was added in a 1:1 stoichiometric ratio (after the induction of ROS treatment). The second group was treated with either **1** or **2** and then H_2_O_2_ was added (prior to ROS induction), and the third group was treated simultaneously with H_2_O_2_ and either **1** or **2** at two distinct stoichiometric ratios (1:5 and 1:1).

#### 4.5.4. Statistical Analysis

The obtained data were presented as average and standard error mean (SEM) values of multiple sets of independent measurements. Mean survival rates and SEMs were calculated for each individual group. Absolute survival rates were calculated for each control group, and one way analysis of variance (ANOVA) was performed for all pair comparisons, followed by post hoc analyses (Tukey) using GraphPad Prism v.6 (GraphPad Software, San Diego, CA, USA). Degrees of significance were assessed by three different rating values: * *p* < 0.05 (significant), ** *p* < 0.01 (highly significant), *** *p* < 0.001 (extremely significant), and **** *p* ≤ 0.0001 (extremely significant), or non-significant (*p* > 0.05).

## 5. Conclusions

The pursuit of enhanced flavonoid functions and new uniquely defined biological antioxidant activities entails a chemical reactivity commensurate with the retention of flavonoid basic core structure and functionality in a cellular setting. To that end, the herein reported work targeted the metal ion complexation of chrysin with the kinetically inert trivalent transition metal ion Cr(III), in hopes of generating well-defined and crystallographically characterized species for a further biological assessment of their antioxidant potency. Consequently, synthetically derived complex materials of Cr(III) and the auxiliary N,N′-aromatic chelators bipy and phen were isolated in crystalline form under varying experimental conditions. The ternary systems exhibit an octahedral environment, bearing replaceable aqua ligands even from a kinetically inert metal ion such as Cr(III), thus (a) signifying the potential chemical reactivity of the modified flavonoid with cellular targets of importance and (b) projecting an alternatively active form of the flavonoid that might exert new and/or enhanced biological activities in biological systems capable of neutralizing ROSs, when the latter emerge under oxidative stress conditions. The physicochemically characterized compounds (**1**,**2**) (via elemental analysis, FT-IR, ESI-MS, luminescence, and X-ray crystallography) formulate a well-defined profile of specifically derivatized flavonoids, further justifying their ensuing investigation in selected biological systems in vitro. The employment of C2C12 cell cultures, exemplifying their diverse biological profile in (patho)physiological processes, led to their exposure to the soluble and bioavailable Cr(III) species and distinctly provided a well-formulated picture of their antiradical potency against oxidative stress conditions. Collectively, both (a) the structural speciation approaches in the synthetic generation of physicochemically well-defined Cr(III)-flavonoid species and (b) the in vitro evaluation of their antioxidant capacity in myoblast cultures exemplify a sustainable enhancement of flavonoid solubility and bioavailability in cellular media that, in the form of metal ion complexation, provide atoxic metal-organic species with measurable antioxidant potency to counteract human pathologies or prevent physiology aberrations. The use of such well-formulated atoxic metal complex flavonoids provides a new impetus into research on antioxidant structure-specific flavonoid-function correlations that might provide a select enhancement of their use in metallodrug formulations counteracting oxidative stress aberrations in human (patho)physiologies.

## Figures and Tables

**Table 1:** Selected geometric parameters (Å, °) in the structures of [Cr(C_15_H_9_O_4_)(C_10_H_8_N_2_)(H_2_O)_2_]_2_(NO_3_)_4_∙3H_2_O (**1**) and [Cr(C_15_H_9_O_4_)(C_12_H_8_N_2_)(H_2_O)(NO_3_)](NO_3_)∙H_2_O (**2**).

Bond Distances (Å)			
Compound 1		Compound 2	
Cr(1)—O(1)	1.9172(18)	Cr(1)—O(1)	1.8911(14)
Cr(1)—O(2)	1.8924(17)	Cr(1)—O(2)	1.9488(14)
Cr(1)—O(5)	1.973(2)	Cr(1)—O(5)	1.9957(18)
Cr(1)—O(6)	1.986(2)	Cr(1)—O(6)	1.9447(18)
Cr(1)—N(1)	2.065(2)	Cr(1)—N(1)	2.0528(18)
Cr(1)—N(2)	2.061(2)	Cr(1)—N(2)	2.0838(17)
**Angles (°)**			
**Compound 1**		**Compound 2**	
O(1)—Cr(1)—O(2)	93.08(7)	O(1)—Cr(1)—O(2)	94.17(6)
O(1)—Cr(1)—O(5)	88.76(9)	O(1)—Cr(1)—O(5)	91.97(7)
O(2)—Cr(1)—O(5)	91.64(9)	O(2)—Cr(1)—O(5)	89.39(7)
O(1)—Cr(1)—O(6)	91.30(9)	O(1)—Cr(1)—O(6)	93.05(7)
O(2)—Cr(1)—O(6)	89.60(8)	O(2)—Cr(1)—O(6)	89.62(7)
O(5)—Cr(1)—O(6)	178.76(8)	O(5)—Cr(1)—O(6)	174.95(7)
O(1)—Cr(1)—N(1)	94.30(8)	O(1)—Cr(1)—N(1)	94.34(7)
O(2)—Cr(1)—N(1)	172.59(8)	O(2)—Cr(1)—N(1)	171.39(7)
O(5)—Cr(1)—N(1)	89.21(9)	O(5)—Cr(1)—N(1)	88.99(8)
O(6)—Cr(1)—N(1)	89.54(8)	O(6)—Cr(1)—N(1)	91.25(8)
O(1)—Cr(1)—N(2)	172.88(8)	O(1)—Cr(1)—N(2)	173.96(7)
O(2)—Cr(1)—N(2)	93.91(8)	O(2)—Cr(1)—N(2)	91.87(6)
O(5)—Cr(1)—N(2)	89.68(9)	O(5)—Cr(1)—N(2)	88.01(7)
O(6)—Cr(1)—N(2)	90.11(8)	O(6)—Cr(1)—N(2)	87.07(7)
N(1)—Cr(1)—N(2)	78.73(9)	N(1)—Cr(1)—N(2)	79.62(7)

**Table 2:** Experimental X-ray crystallographic details on the compounds [Cr(C_15_H_9_O_4_)(C_10_H_8_N_2_)(H_2_O)_2_]_2_(NO_3_)_4_∙3H_2_O (**1**) and [Cr(C_15_H_9_O_4_)(C_12_H_8_N_2_)(H_2_O)(NO_3_)](NO_3_)∙H_2_O (**2**).

Crystal Data	Compound 1	Compound 2
Chemical formula	C_25_H_24_CrN_4_O_13.50_	C_27_H_21_CrN_4_O_12_
*M*_r_	648.48	645.48
Crystal system	Triclinic	Monoclinic
Space group	*P*ī	*P*2_1_/*n*
Temperature (K)	295	295
*a* (Å)	8.3924 (8)	12.5502 (17)
*b* (Å)	11.1091 (11)	18.337 (2)
*c* (Å)	16.7214 (15)	13.5063 (18)
α (°)	91.576 (3)	90
β (°)	101.702 (3)	103.644 (4)
γ (°)	92.931 (3)	90
*V* (Å^3^)	1523.4 (3)	3020.6 (7)
*Z*	2	4
Radiation type	Mo *K*α	Mo *K*α
μ (mm^−1^)	0.45	0.45
Crystal size (mm)	0.15 × 0.12 × 0.08	0.24 × 0.22 × 0.10
**Data Collection**		
Diffractometer	Bruker Kappa Apex2	
Absorption correction	Numerical	
*T*_min_, *T*_max_	0.95, 0.96	0.91, 0.96
**Reflections**, number of		
measured	26,395	37,384
independent	5916	5729
observed [*I* > 2.0σ(*I*)]	4343	4584
*R*_int_	0.022	0.018
(sin θ/λ)_max_ (Å^−1^)	0.617	0.611
**Refinement**		
*R*[*F*^2^ > 2σ(*F*^2^)]	0.043	0.043
*R_w_*(*F*^2^)	0.103	0.062
*S*	1	1
No. of reflections	4343	4584
No. of parameters	404	393
No. of restraints	6	17
H-atom treatment	H-atom parameters constrained	
Δρ_max_, Δρ_min_ (e Å^−3^)	0.38, −0.90	0.43, −0.25

## Data Availability

No data.

## Supplementary Materials

The following supporting information can be downloaded at: https://www.mdpi.com/article/10.3390/ijms23137171/s1.

## Abbreviations

ANOVA	one-way analysis of variance
DMEM	Dulbecco’s modified Eagle’s medium
ESI-MS	electron spray ionization–mass spectrometry
FBS	fetal bovine serum
FT-IR	Fourier transform infrared
ROS	reactive oxygen species
SEM	standard error of the mean
XTT	sodium 3-[1-(phenylaminocarbonyl)-3,4-tetrazolium]-bis(4-methoxy-6-nitro) benzenesulfonic acid hydrate
