# Supplementary material for: Chromium Flavonoid Complexation in an Antioxidant Capacity Role
Source: Int J Mol Sci. 2022 Jun 28;23(13):7171. doi: 10.3390/ijms23137171 (PMC9266733; doi:10.3390/ijms23137171)
Supplement: Supplementary file 1 [file ijms-23-07171-s001.zip › ijms-1708316-supplementary.pdf]

Chromium flavonoid complexation in an antioxidant capacity role

S. Matsia,[a] O. Tsave,[a] A. Hatzidimitriou,[b] A. Salifoglou[a]*

* Author to whom correspondence should be addressed.

Tel: +30-2310-996-179, E-mail: salif@auth.gr

[a] Laboratory of Inorganic Chemistry and Advanced Materials, School of Chemical Engineering, Aristotle University of Thessaloniki, Thessaloniki 54124, Greece

[b] Laboratory of Inorganic Chemistry, School of Chemistry, Aristotle University of Thessaloniki, Thessaloniki 54124, Greece

Tables

Table S1. Hydrogen-bond geometry (Å, º)

Compound 1

D—H···*A*	*D*—H	H···*A*	*D*···*A*	*D*—H···*A*
O5—H51···O10	0.82	1.80	2.618 (4)	174
O5—H52···O9^{i}	0.82	1.88	2.694 (4)	174
O6—H61···O7	0.82	1.92	2.741 (4)	177
O6—H62···O17^{ii}	0.82	1.74	2.549 (4)	173
O6—H62···N5^{ii}	0.82	2.51	3.293 (4)	160
O4—H253···O8^{v}	0.82	2.55	3.101 (4)	125
O4—H253···O9^{v}	0.82	1.94	2.760 (4)	179
O10—H101···O4^{i}	0.83	2.04	2.867 (4)	175
O10—H102···O13	0.82	1.99	2.808 (4)	174
O14—H254···O17^{ii}	0.82	2.23	2.895 (4)	139
O14—H142···O16^{ii}	0.82	1.53	2.138 (4)	128

Symmetry codes: (i) *x*+1, *y*, *z*; (ii) *x*, *y*−1, *z*; (v) −*x*, −*y*+1, −*z*+1.

Compound 2

D—H···*A*	*D*—H	H···*A*	*D*···*A*	*D*—H···*A*
O4—H42···O12^{ii}	0.82	2.33	3.149 (3)	180
O4—H42···O13^{ii}	0.82	2.55	3.122 (3)	128
O5—H51···N4	0.82	2.51	3.279 (3)	157
O5—H51···O12	0.82	1.59	2.406 (3)	173
O5—H52···N3	0.82	2.49	3.246 (3)	154
O5—H52···O9^{i}	0.82	2.49	3.031 (3)	124
O5—H52···O10	0.82	1.90	2.715 (3)	176
O14—H142···O15^{i}	0.82	1.71	2.360 (3)	134
O14—H143···O11^{iv}	0.82	2.45	3.245 (3)	163
O14—H143···O13^{iv}	0.82	2.22	2.925 (3)	144
O15—H152···O7^{v}	0.82	2.31	3.117 (3)	168
O15—H252···O10	0.82	2.12	2.937 (3)	178

Symmetry codes: (i) −*x*+1, −*y*+1, −z+2; (ii) −*x*+1/2, *y*−1/2, −z+3/2; (iv) −*x*, −*y*+1, −z+2; (v) −*x*+1, −*y*+1, −z+1.

Table S2. Molecular absorption coefficient of compounds **1-2** in methanol and DMSO

Solvent	Compounds	Wavelength (λ_{max}, nm)	Molecular Absorption Coefficient (ε, M^{-1}•cm^{-1})
**Methanol**	**1**	399	5,806
		341	11,091
		293	32,194
		206	60,179
	**2**	400	5,660
		341	10,559
		274	58,423
		206	123,429
**DMSO**	**1**	400	6,490
		341	13,167
		298	36,390
	**2**	402	6,516
		341	13,325
		276	40,650

Figure captions

Figure S1: **(A).** Comparative FT-IR spectra of Cr-chr-bipy (compound **1**) with chr recorded in KBr.

(**B).** Comparative FT-IR spectra of Cr-chr-phen (compound **2**) with chr recorded in KBr.

Figure S2: Full ESI-MS spectra of compound **1** recorded in methanol upon positive ionization

Figure S3: Full ESI-MS spectra of compound **2** recorded in methanol upon positive ionization

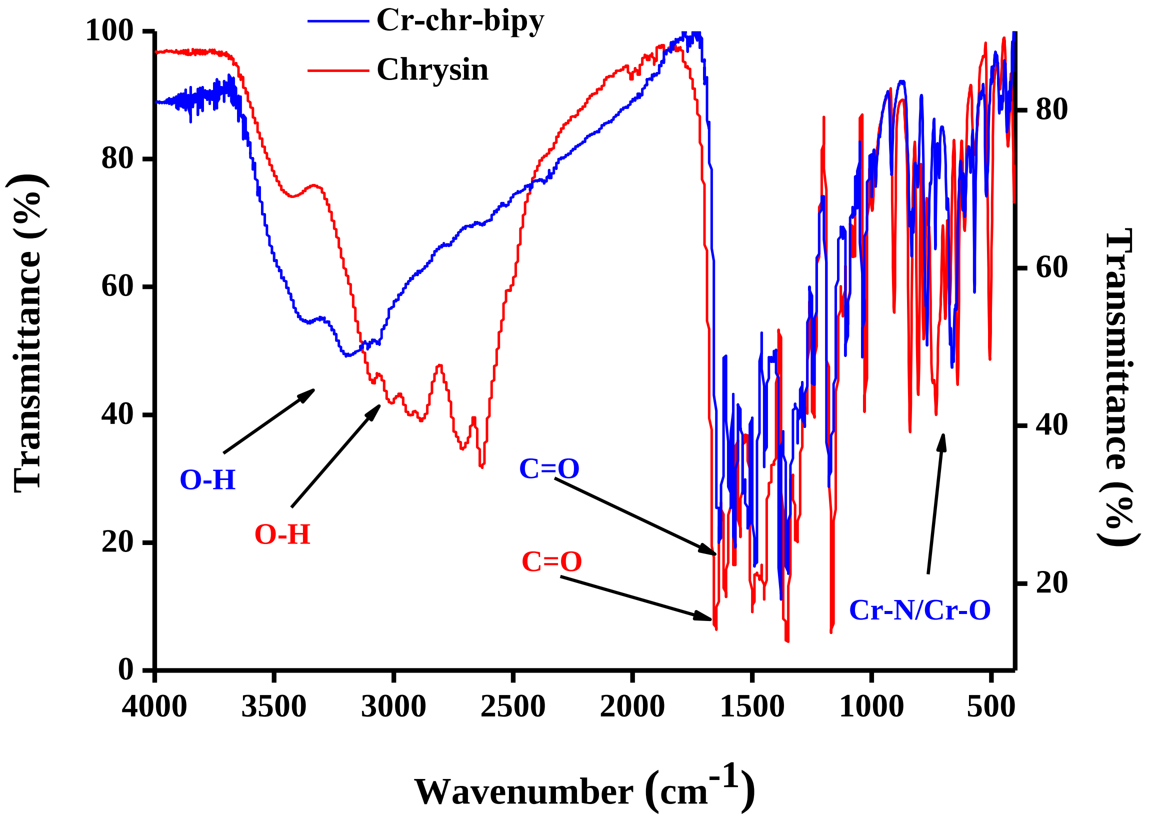
Cr-chr-bipy
Chrysin
Transmittance (%)
Transmittance (%)
Wavenumber (cm^{-1})
O-H
O-H
C=O
C=O
Cr-N/Cr-O
100
80
60
40
20
0
80
60
40
20
4000
3500
3000
2500
2000
1500
1000
500

Figure S1A

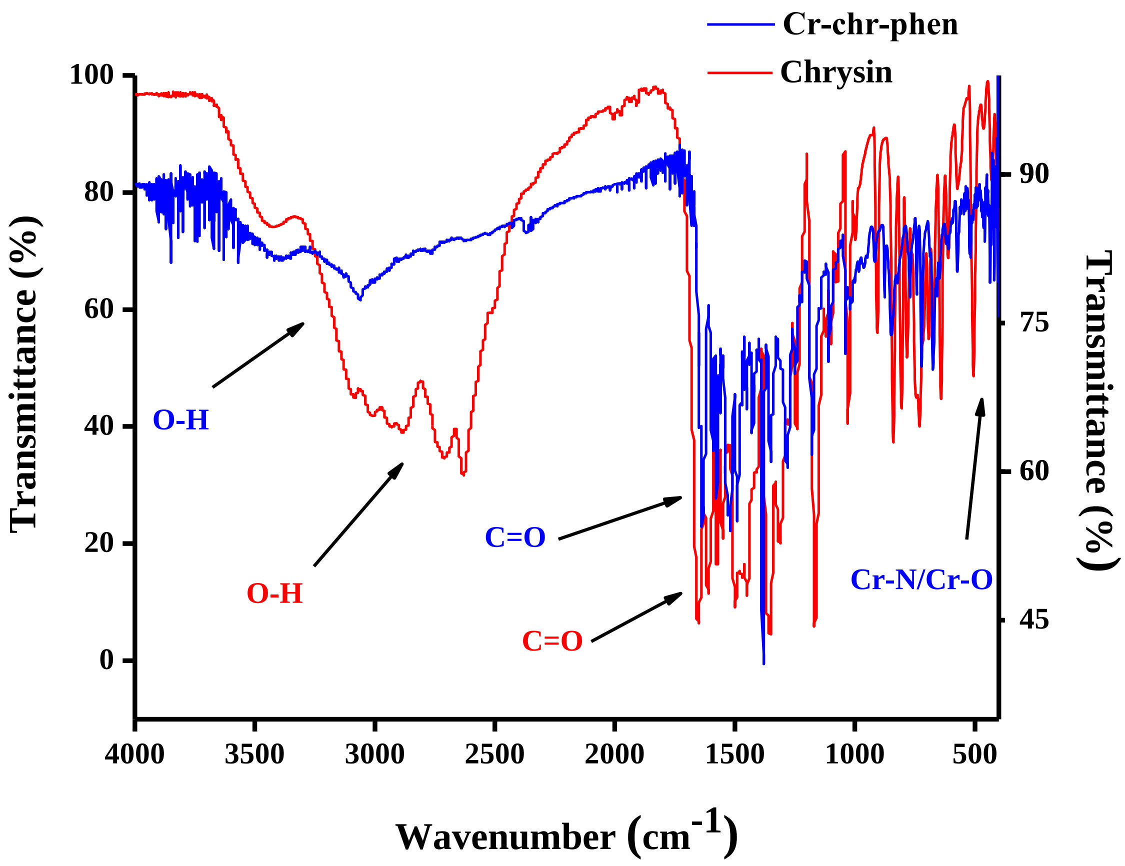
Cr-chr-phen
Chrysin
Transmittance (%)
100
80
60
40
20
0
O-H
O-H
C=O
C=O
Cr-N/Cr-O
90
75
60
45
Transmittance (%)
4000
3500
3000
2500
2000
1500
1000
500
Wavenumber (cm-1)

Figure S1B

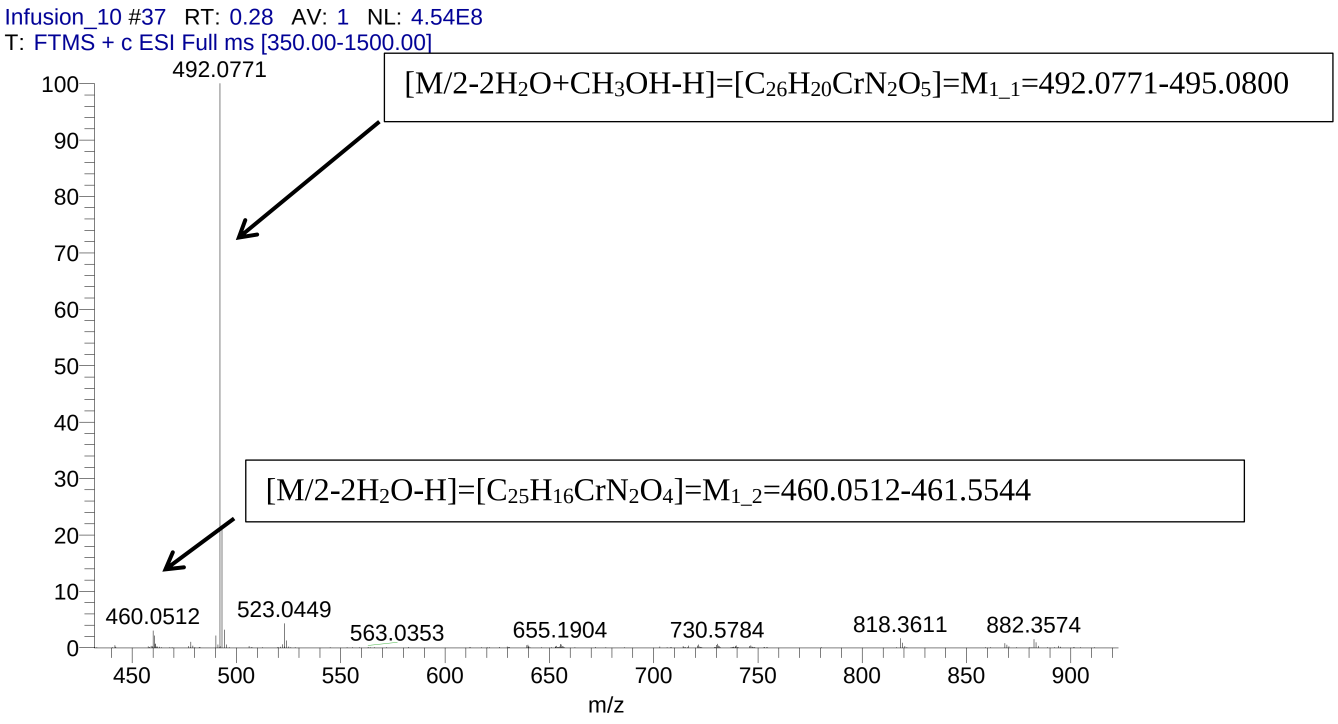
Infusion_10 #37 RT: 0.28 AV: 1 NL: 4.54E8
T: FTMS + c ESI Full ms [350.00-1500.00]
492.0771
[M/2-2H2O+CH3OH-H]=[C26H20CrN2O5]=M1_1=492.0771-495.0800
[M/2-2H2O-H]=[C25H16CrN2O4]=M1_2=460.0512-461.5544
460.0512
523.0449
563.0353
655.1904
730.5784
818.3611
882.3574
m/z

Figure S2

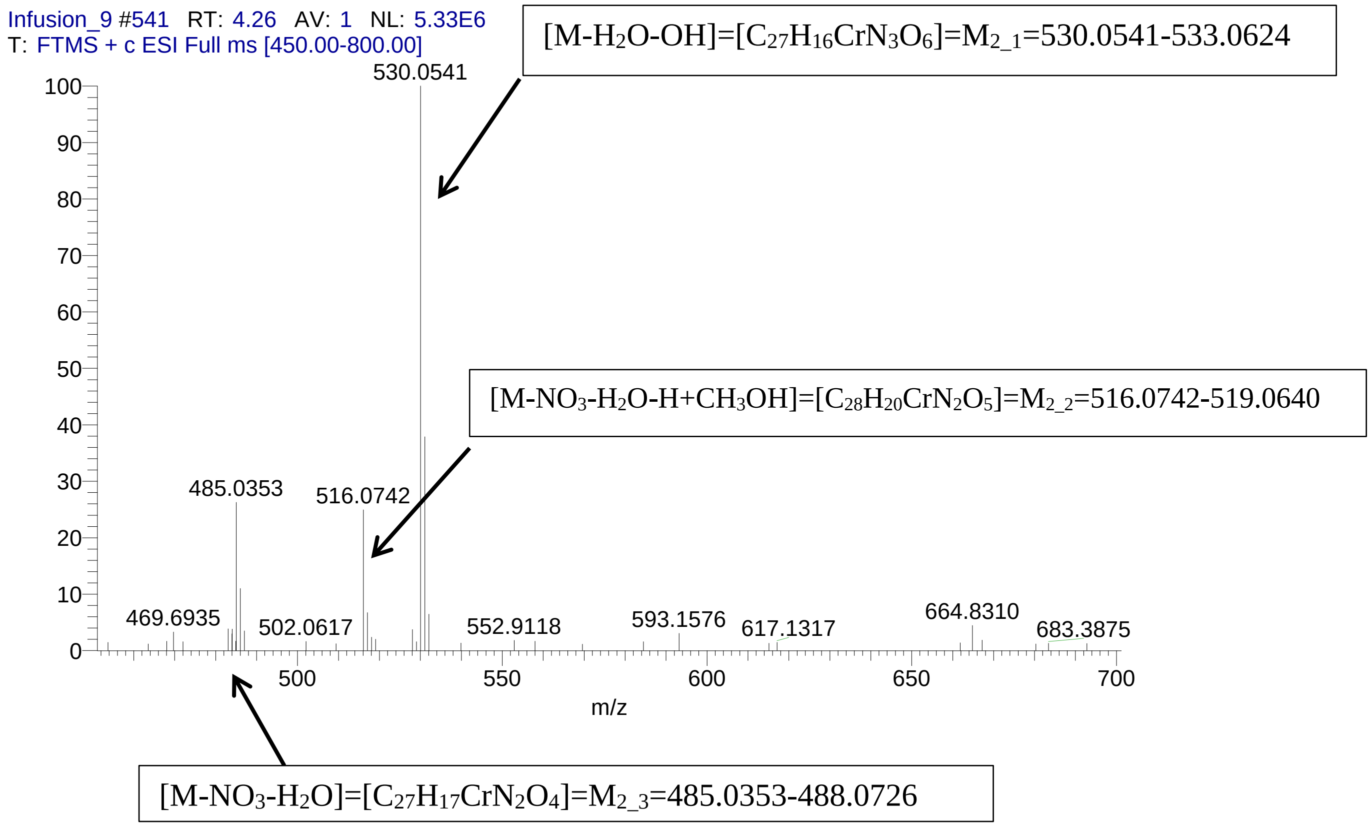
Infusion_9 #541 RT: 4.26 AV: 1 NL: 5.33E6
T: FTMS + c ESI Full ms [450.00-800.00]
[M-H2O-OH]=[C27H16CrN3O6]=M2_1=530.0541-533.0624
[M-NO3-H2O-H+CH3OH]=[C28H20CrN2O5]=M2_2=516.0742-519.0640
[M-NO3-H2O]=[C27H17CrN2O4]=M2_3=485.0353-488.0726
530.0541
485.0353
516.0742
469.6935
502.0617
552.9118
593.1576
617.1317
664.8310
683.3875
m/z

Figure S3
